# Quantitative Hormone Signaling Output Analyses of *Arabidopsis thaliana* Interactions With Virulent and Avirulent *Hyaloperonospora arabidopsidis* Isolates at Single-Cell Resolution

**DOI:** 10.3389/fpls.2020.603693

**Published:** 2020-11-06

**Authors:** Hassan Ghareeb, Mohamed El-Sayed, Michael Pound, Olena Tetyuk, Katharina Hanika, Cornelia Herrfurth, Ivo Feussner, Volker Lipka

**Affiliations:** ^1^Department of Plant Cell Biology, Albrecht-von-Haller Institute of Plant Sciences, University of Göttingen, Göttingen, Germany; ^2^Department of Plant Biotechnology, National Research Centre, Cairo, Egypt; ^3^School of Computer Science, University of Nottingham, Nottingham, United Kingdom; ^4^Department of Plant Biochemistry, Albrecht-von-Haller Institute of Plant Sciences, University of Göttingen, Göttingen, Germany; ^5^Service Unit for Metabolomics and Lipidomics, Göttingen Center for Molecular Biosciences (GZMB), University of Göttingen, Göttingen, Germany; ^6^Central Microscopy Facility of the Faculty of Biology and Psychology, University of Göttingen, Göttingen, Germany

**Keywords:** ethylene, intercellular communication, jasmonate, plant immunity, plant-pathogen interactions, signaling output reporters, salicylic acid, single-cell resolution

## Abstract

The phytohormones salicylic acid (SA), jasmonic acid (JA), and ethylene (ET) are central regulators of biotic and abiotic stress responses in *Arabidopsis thaliana*. Here, we generated modular fluorescent protein-based reporter lines termed COLORFUL-PR1pro, -VSP2pro, and -PDF1.2apro. These feature hormone-controlled nucleus-targeted transcriptional output sensors and the simultaneous constitutive expression of spectrally separated nuclear reference and plasma membrane-localized reporters. This set-up allowed the study of cell-type specific hormone activities, cellular viability and microbial invasion. Moreover, we developed a software-supported high-throughput confocal microscopy imaging protocol for output quantification to resolve the spatio-temporal dynamics of respective hormonal signaling activities at single-cell resolution. Proof-of-principle analyses in *A. thaliana* leaves revealed distinguished hormone sensitivities in mesophyll, epidermal pavement and stomatal guard cells, suggesting cell type-specific regulatory protein activities. In plant-microbe interaction studies, we found that virulent and avirulent *Hyaloperonospora arabidopsidis* (*Hpa*) isolates exhibit different invasion dynamics and induce spatio-temporally distinct hormonal activity signatures. On the cellular level, these hormone-controlled reporter signatures demarcate the nascent sites of *Hpa* entry and progression, and highlight initiation, transduction and local containment of immune signals.

## Introduction

The phytohormones salicylic acid (SA), jasmonic acid (JA), and ethylene (ET) represent fundamental components of the plant immune signaling network (Robert-Seilaniantz et al., 2011). Pathogen attack alters SA, JA, and ET homeostasis in the challenged plant either leading to activation or suppression of innate immunity (Pieterse et al., 2009; Kazan and Lyons, 2014). SA signaling is indispensable for defense against biotrophic plant pathogens (Tsuda and Katagiri, 2010; Caillaud et al., 2013; Liu et al., 2016; Ding et al., 2018) and is characterized by reciprocal antagonism with the JA pathway (Thaler et al., 2012). Regulation of SA-induced responses is controlled by NON-EXPRESSOR OF PATHOGENESIS-RELATED GENES 1 (NPR1), which operates as transcriptional co-activator of target genes including the marker *PATHOGENESIS-RELATED GENE 1* (*PR1*) (Ding et al., 2018). Moreover, NPR1 functions as a canonical crosstalk modulator between the SA, JA and ET signaling pathways (Leon-Reyes et al., 2009; Broekgaarden et al., 2015). JA triggers expression of the marker gene *VEGETATIVE STORAGE PROTEIN* 2 (*VSP2*) (Chini et al., 2007; Mousavi et al., 2013), but also acts in combination with ET (JA/ET) to control a distinct downstream response characterized by upregulation of *PLANT DEFENSIN 1.2a* (*PDF1.2a*) gene expression (Van der Does et al., 2013). Whilst JA and ET have been demonstrated to synergistically regulate defense against necrotrophic pathogens (Zhu, 2014), the marker gene-defined JA and JA/ET signaling pathways can act antagonistically upon each other (Lorenzo et al., 2004), and are suppressed by SA (Van der Does et al., 2013). These findings underpin the complex crosstalk between these hormone signals and their importance for coordinating and fine-tuning defense against different types of pathogens (Thomma et al., 1998; Robert-Seilaniantz et al., 2011).

The ultimate outcome of plant-pathogen interactions is predetermined at the initial site of attack, where the pathogen invades a single or few cell(s) of the host. This is particularly obvious in the exemplary compatible and incompatible interactions between the biotrophic oomycete pathogen *Hyaloperonospora arabidopsidis* (*Hpa*) and the plant model *A. thaliana* (Coates and Beynon, 2010). In compatible interactions, virulent *Hpa* isolates penetrate the host leaf surface, invade anticlinal cell walls between neighboring epidermal cells, proliferate and establish haustoria in individual epidermal pavement and underneath mesophyll cells (Coates and Beynon, 2010). Recognition of the oomycete through pathogen-associated molecular patterns (PAMPs) activates the first layer of the plant immune system, so-called PAMP-triggered immunity (PTI) (Fabro et al., 2011). Effector-mediated suppression of PTI allows further proliferation, colonization of the entire leaf and completion of the pathogen life cycle (Caillaud et al., 2013; Deb et al., 2018). In incompatible interactions with avirulent *Hpa* isolates, recognition of effectors activates a second defense mode, designated effector-triggered immunity (ETI), which is typically associated with hypersensitive response (HR)-like cell death and restriction of pathogen growth to the initially invaded plant cells (van der Biezen et al., 2002; Wang et al., 2011). Conceivably, in order to execute this locally confined response plants must have a highly coordinated and spatio-temporally controlled immune signaling system.

To investigate the role of SA, JA, and ET in regulating immune responses against *Hpa*, several approaches, such as hormone quantification, measurement of marker gene expression as well as mutant analysis have been used (Wang et al., 2011; Murmu et al., 2014; Wirthmueller et al., 2018). However, these approaches are invasive and largely constrained to organ and tissue levels. To gain insights into SA, JA, and ET signaling dynamics during plant-pathogen interactions at cellular resolution, quantitative tools allowing studies on cellular activity and crosstalk are required. Here, we describe the development of a tri-modular reporter system that we used to reveal so far unknown spatio-temporal signatures of SA, JA, and JA/ET action stimulated by hormone treatment or during compatible and incompatible *A. thaliana-Hpa* interactions at the single-cell level.

## Materials and Methods

### Plant Materials and Growth Conditions

*A. thaliana* (Col-0) was used for *Agrobacterium*-mediated transformation of the reporter constructs, and *npr1*-1 (Cao et al., 1994), *coi1*-t (Mosblech et al., 2011), and *ein2*-1 (Guzman and Ecker, 1990) mutants were used for crossings.

For pathogen inoculation experiments, seeds of *A. thaliana* (Col-0), or T4 generations of the reporter lines containing single T-DNA insertions of the respective COLORFUL constructs were sown onto 8 cm^2^ pots and vernalized at 4°C. Seedlings were grown in climate chambers (Johnson Controls, United States) under short day conditions (8 h light/16 h dark) with 150 μmol⋅m^–2^⋅s^–1^ at 22°C/18°C for a week and transplanted into individual pots for an additional 2 weeks.

For hormone treatments, the seeds were sterilized with 99% ethanol for 1 min and with 70% ethanol containing 0.05% Tween20 for 5 min on a rotator (20 rpm). Seeds were washed twice with sterilized distilled water and suspended in sterilized 0.1% agarose. Seeds were stratified at 4°C for 72 h and then sown onto 1/2 MS/MES agar plates (MS 2.2 g/l, MES 0.5 g/l, plant agar 7 g/l, pH 5.8). Seedlings were grown for 11 days in a climate chamber under long day conditions (16 h light/8 h dark) with 150 μmol⋅m^–2^⋅s^–1^ at 22°C/18°C.

### Plasmid Constructions

The individual modules of the COLORFUL reporters were generated using the COLORFUL-Circuit vector system (Ghareeb et al., 2016). The VENUS-N7 fragment was amplified with PCR from the plasmid pENTR-*VENUS-N7* using the oligonucleotide primers oHG19 (5′-TAGC TGGATCCTGTATGGTGAGCAAGGGCGAGGAGC-3′) and oHG20 (5′ - GGCCGACTAGTATTACTCTTCTTCTTGAT CAGC-3′) and then cloned in the plasmid pC3 (Ghareeb et al., 2016) using *Bam*HI and *Spe*I sites to generate pC3-*UBQ10-VENUS-N7*. For promoter module construction, 1.35, 1, and 1.1 kb fragments upstream of the translational initiation sites of *PR1*, *VSP2* and *PDF1.2a*, respectively, were PCR-amplified from genomic DNA of ecotype Col-0 using the primer pairs oHG26 (5′-GCGG CCGGTCCGACGTAATAATATCCTATGGTG-3′) and oHG 27 (5′-GGCGCCGGACCGTTTTCTAAGTTGATAATGGTTA TTG-3′), oHG24 (5′-GTAGCCGGTCCGAAACCGTCGAAAAT TTTCGACCG-3′) and oHG25 (5′-GGCCGCCGGACCGGT TTTTTATGGTATGGTTTATTG-3′), and oHG73 (5′-ATAT AGGCCAGTCTGGCCCGGTCCGCCAAAGCAAAAGTTCTA AGCCC-3′) and oHG74 (5′-GCCGGCGGACCGGATGA TTATTACTATTTTGTTTTC-3′). The PCR products were used to replace the UBQ10 promoter using *Rsr*II sites in pC3-*UBQ10-VENUS-N7* to generate the plasmids pC3-*PR1-VENUS-N7*, pC3-*VSP2-VENUS-N7* and pC3-*PDF1.2a-VENUS-N7*, respectively. For reference expression cassette generation, *mKATE2* was amplified from pC1 (Ghareeb et al., 2016) using the primers oHG11 (5′-TAGCTGGATCCATGGTGAGCGAGCTGATTAAGG-3′) and oHG29 (5′-GGGCTGAATTCCAATTCTGTGCCCCAGTT TGC-3′) and replaced *VENUS* in pC3-*UBQ10-VENUS-N7* using *Bam*HI and *Eco*RI sites to generate pC3-*UBQ10-mKATE2-N7*. The *35S-EGFP-LTI6b* and *UBQ10-mKATE2-N7* gene cassettes were amplified from the plasmids pEGFP-LTI6b (Federici et al., 2012) and pC3-*UBQ10-mKATE2-N7* using the primer pairs oHG122 (5′-TCTGGGGACCTGCAGGCATGGGCCGGTCACTGGATT TTGG-3′) and oHG79 (5′-ATATTGGCCTCTGTGGCCTCA TCAACCAGCGGAAGCGG-3′), and oHG76 (5′-CATGCCTGC AGGTCCCCAGATTAGCCTTTTC-3′) and oHG80 (5′-ACC TAGGCCTGGTTGGCCTTCATGTTCTTTCCTGCG-3′) and cloned in pC2 (Ghareeb et al., 2016) using *Sfi*I and *Pst*I sites to generate the plasmid pC2-*UBQ10-mKATE2-N7*/*35S-EGFP-LTI6b*. *PR1-VENUS-N7*, *VSP2-VENUS-N7* or *PDF1.2a-VENUS-N7*, and *UBQ10-mKATE2-N7*/*35S-EGFP-LTI6b* were excised with *Sfi*I from the aforementioned plasmids and assembled in a *Sfi*I-digested pC2-C3 binary vector (Ghareeb et al., 2016) to produce the pCOLORFUL-PR1pro, pCOLORFUL-VSP2pro and pCOLORFUL-PDF1.2apro vectors, respectively. All plasmid constructs generated in this study will be made available upon request.

### *A. thaliana* Transformation

Stable plant transformation of *A. thaliana* plants was performed using floral dipping according to Ghareeb et al. (2016). Eight pots containing five plants were grown under long day conditions. Five weeks post germination (wpg), shoots of the first inflorescence were excised. *Agrobacterium tumefaciens* GV3101/pSoup strains containing the corresponding reporter plasmid were grown in LB liquid medium containing 30 mg/l gentamicin, 50 mg/l rifampicin, 50 mg/l kanamycin, 2.5 mg/l tetracycline. Grown cultures were inoculated in 500 ml of LB liquid medium supplemented with the same antibiotic concentrations and incubated overnight. Pellets of these bacterial cultures were resuspended in 500 ml infiltration medium (2.2 g/l MS with Gamborg B5 vitamins, 50 g/l sucrose, and 150 μl/l Silwet L-77). The plants were dipped at six wpg and incubated overnight in plastic bags in the dark. Five days later floral dipping was repeated. Seeds were collected 4 weeks later. Transformants were isolated by repeated spraying with 0.1% Basta (Bayer CropScience, Germany) 1 wpg. All transgenic lines generated in this study will be made available upon request.

### Hormone Treatments

Hormone treatments were performed as previously reported (Van der Does et al., 2013). Twelve day-old seedlings were incubated in 24 multiwell plate containing 1.5 ml KCl/MES buffer (1 mM KCl and 5 mM MES, pH 5.8) and after 24 h the mock seedlings were treated with 0.5 ml KCl/MES buffer containing the corresponding amount of solvent used for hormone preparation. For hormone treated seedlings, 0.5 ml KCl/MES buffer supplemented with a 4-fold hormone concentration was applied. Hormone stock solution of 0.5 M sodium salicylate (Sigma-Aldrich) dissolved in water, 100 mM MeJA (95%, Sigma-Aldrich) dissolved in 99% ethanol, and 200 mM ACC (Calbiochem) dissolved in water were used to prepare the final hormone concentrations.

### Inoculation With *Hpa*

The spores of the *Hpa* Noco2 and Emwa1 isolates were collected in water by gently shaking of infected *A. thaliana* seedlings, respectively. Three weeks old plants were sprayed with 5 × 10^4^ spores ml^–1^, and then covered with a heavy transparent cover to maintain high humidity. Afterward the plants were transferred to a growth chamber with short day conditions (9 h light/15 h dark), 150 μmol⋅m^–2^⋅s^–1^ at 17°C and 92/98% relative humidity.

### Confocal Laser Scanning Microscopy

Imaging was performed using TSC-SP5 and TSC-SP8 laser scanning confocal microscopes (Leica, Bensheim, Germany) in 8-bit formats. For image generation, the HCX PL APO CS 40.0 × 0.70 dry and HC PL APO CS2 20.0 × 0.75 oil-immersion objective, sequential and bidirectional scan with a speed of 200 Hz were used. Images were acquired with 2×, 3×, and 2× line averages for the VENUS, EGFP, and mKATE2 channels, respectively. For fluorescence quantification purposes, the HCX PL APO CS 20.0 × 0.70 dry objective with 2× zoom factor, sequential and bidirectional scan with a speed of 400 Hz, a single line averaging, and a resolution of 512 × 512 pixels were used. Fluorescent Brightener 28 was excited using 405 nm UV diode laser. EGFP and VENUS were excited with the 488 and 514 nm lines of an argon ion laser, respectively, whereas mKATE2 was excited with a 594 nm HeNe laser. The emission spectra were set to 420–460 nm for Fluorescent Brightener 28, 492–510 nm for EGFP, 528–555 nm for VENUS, and 610–640 nm for mKATE2. Emitted fluorescence was configured to pass through pinhole aperture of 1 airy unit and detected with HyD detectors.

The 3rd leaf from hormone-treated seedlings or the 5th leaf from *Hpa*-inoculated plants was selected for microscopy. For the *Hpa*-inoculated samples, the adaxial surface was emerged in a drop of 10 μg/ml Fluorescent Brightener 28 (Sigma-Aldrich, Taufkirchen, Germany) for 30 s to stain the *Hpa* spores. The leaf midrib was excised, the leaf placed on 3 well-containing slide (Thermo Fisher Scientific, Braunschweig, Germany) and mounted in the Fluorescent Brightener 28. The EGFP and Fluorescent Brightener 28 channels were used to define individual sites of *Hpa* attack. The mKATE2 channel was used to assign the z-stack start and end planes for optimal coverage of the nuclei-localized fluorescence signals. The VENUS signal in the treated samples was used to set the threshold of fluorescence detection in comparison to mock. Saturation of fluorescence signals was avoided. The same imaging configurations were applied for mock and treatment. The maximum intensity projections, contrast and image merging were performed using Fjji (ImageJ v1.51).

### Cellular Fluorescence Quantification

COLORFUL SPOTTER is written in Java as a plugin for ImageJ (Schindelin et al., 2012), and is designed to calculate the intensity levels of nuclear markers throughout multiple images. The plugin is open-source and available at https://github.com/mikepound/colorful. Image processing occurs automatically over a directory of input images, with an additional graphical interface to define the reference and reporter channels. Before detection of the nuclei within each image, a median filter is used to remove common grainy noise without affecting the boundary positions of the nuclei. Once filtered, the plugin extracts nuclei locations from the reference image by default through Otsu thresholding algorithm, which calculates an optimal threshold by maximizing the intra-class variance of the background and foreground. Optionally, additional input channels are added to the reference channel by performing a pixel-wise sum operation. All foreground pixels are then determined by automatic thresholding over the summed image.

Nuclei are detected within the thresholded image using a 4-way connected component algorithm adopted from the single-pass approach taken in (Blob Labeller, ImageJ plugin). Small noise artifacts (3 pixels or fewer) are removed by default, to avoid false positives. Each nucleus is assigned a unique index, and its area is measured. The mean intensity of the reference and reporter, and their ratio within the nucleus is quantified. The results are automatically exported as an Excel compatible file as well as images depicting all detected nuclei and their indexes, usable for quality control analysis and manual sorting of single-cell readouts.

### Statistical Analysis

All presented data were obtained from independent biological replicates (plants). For each plant a single confocal microscopy image was captured. Cell type-specific single cell readouts from individual images were averaged and represent a single replicate. For results presentation, relative values to the mean of the corresponding mock of the same cell type were calculated. Next, the data was log transformed. Normality was tested using boxplots, whenever *n* was more than 6. The assumption of variance homogeneity was tested using the Levene’s test and boxplot of the residuals. For testing statistical significance of differences, Student’s *t*-test, and one-way and two-way ANOVA followed with Tukey test for multiple comparison analysis were independently performed for the log-transformed data using the R software. Student’s *t*-test was used to test the significance difference of a treated sample to the corresponding mock, and the ANOVA and Tukey tests were used to analyze the significance difference within all groups.

### Transcript Expression Analysis

Total RNA was extracted according to Ghareeb et al. (2011). RNA was reverse transcribed using SuperScript III First-Strand Synthesis System (Thermo Fisher Scientific). SsoFast EvaGreen Supermix (Bio-Rad) and the *PR1* primers oHG239 (5′-TTCTTCCCTCGAAAGCTCAA-3′) and oHG240 (5′-AAGGCCCACCAGAGTGTATG-3′), *VSP2* primers oHG148 (5′-CGCAAAATATGGATACGGAAC-3′) and oHG149 (5′-GACATTCTTCCACAACTTCC-3′), PDF1.2 primers oHG146 (5′-CTTGTTCTCTTTGCTGCTTTC-3′) and oHG147 (5′-CATGTTTGGCTCCTTCAAG-3′), and UBQ5 (internal control) primers oHG150 (5′-GACGCTTCATCTCGTCC-3′) and oHG151 (5′-GTAAACGTAGGTGAGTCCA-3′) were used for quantification of the transcript abundance. Transcript levels were calculated relative to the internal control.

### Determination of Phytohormones by UPLC-Nano ESI-MS/MS

Three-week-old Col-0 plants were spray-inoculated with water (mock), or *Hpa* conidiospores, and the 5th leaves from 40 to 45 plants per treatment were collected at 1 and 2 dpi. Phytohormones were extracted with methyl-*tert*-butyl ether (MTBE), reversed phase-separated using an ACQUITY UPLC system (Waters Corp., Milford, MA, United States) and analyzed by nanoelectrospray ionization (nanoESI) (TriVersa Nanomate; Advion BioSciences, Ithaca, NY, United States) coupled with an AB Sciex 4000 QTRAP tandem mass spectrometer (AB Sciex, Framingham, MA, United States) employed in scheduled multiple reaction monitoring mode (for details see Supplementary Methods S1).

## Results

### COLORFUL-PR1pro Reveals SA-Dependent Signaling Outputs at Single-Cell Resolution

**FIGURE 1:**
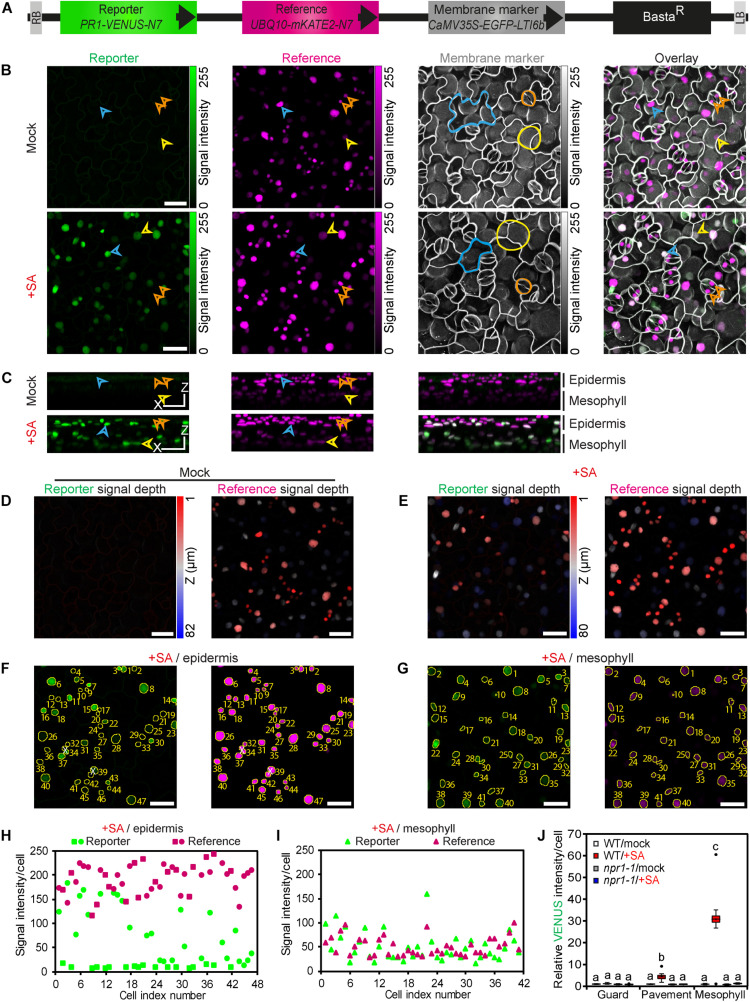
*A. thaliana* COLORFUL-PR1pro reporter lines enable qualitative and quantitative monitoring of SA signaling responses at single-cell resolution. **(A)** Schematic representation of the COLORFUL-PR1pro T-DNA construct harboring the fluorescent modules PR1-VENUS-N7 (nuclear localized reporter), UBQ10-mKATE2-N7 (nuclear-localized reference) and CaMV35S-EGFP-LTI6b (plasma membrane marker). Basta^*R*^, selectable marker; LB/RB, left/right borders of T-DNA. **(B)** Maximum projections of CLSM z-stack images showing expression of reporter modules in leaves of 12-day-old transgenic *A. thaliana* line COLORFUL-PR1pro#1 at 24 h post incubation with mock solution (top) or 0.5 mM sodium salicylate (bottom); epidermal cells (cyan arrowhead/line), mesophyll cells (yellow arrowhead/line) and guard cells (orange arrowhead/line). **(C)** Side-view 3D projections of the CLSM z-stack images presented in b show epidermis and mesophyll cell-specific distribution and intensities of reporter- and reference-associated fluorescence signals (left and middle) and their overlays (right). XZ Scale bar = 25 μm. **(D,E)** z-depth of reporter (left) and reference (right) fluorescence signals from mock **(D)** and SA **(E)** treatments shown in b. Scale bar, 25 μm. **(F,G)** Automated nuclear fluorescence detection and quantification using the COLORFUL SPOTTER plugin. CLSM z-stack images corresponding to epidermal **(F)** and mesophyll **(G)** cell layers from the SA treatment experiment presented in B (bottom) were used separately for generation of tissue-specific maximum projections of reporter (left) or reference (right) fluorescence. Subsequent COLORFUL SPOTTER-mediated processing identifies nuclear fluorescence signals, generates a corresponding fluorescence detection mask (yellow overlay), assigns cell-specific index numbers, and quantifies individual fluorescence intensities **(H,I)**. White X indicates manually excluded spots resulting from erroneous merging of close nuclear signals. Scale bar, 25 μm. **(H,I**), COLORFUL SPOTTER-mediated quantification of single-cell reporter and reference fluorescence signals from f and g, respectively. **(J)** Reporter activities in leaf guard, pavement and palisade mesophyll cells of wild-type (WT) and *npr1-1* mutant plants after treatment of seedlings with 0.5 mM sodium salicylate for 24 h. Box plots show first quartile (lower line); median (center line); mean (+); third quartile (upper line); whiskers extend 1.5 times the interquartile range, and outliers (dots), *n* = 9–10. Data are relative to mock of WT. Different letters indicate significant differences (Two-way ANOVA followed by Tukey’s multiple comparison test, *p* < 0.05; see also Supplementary Figures S1, S2A, S4A).

To reveal the spatial dynamics of SA-dependent signaling processes at single-cell resolution, we developed a confocal laser scanning microscopy (CLSM)-based reporter system in *A. thaliana* plants. To this end, we utilized our recently published multigene assembly vector platform “COLORFUL-Circuit” (Ghareeb et al., 2016), and combined three distinct fluorescent protein-based reporter cassettes and a BASTA-resistance selection marker on a single binary vector for one-step simultaneous transgenic expression (COLORFUL-PR1pro; Figure 1A). The three reporter modules employ the spectrally separable and monomeric fluorescent proteins VENUS, mKATE2 and EGFP (Zhang et al., 1996; Nagai et al., 2002; Shcherbo et al., 2009), whose expression are controlled by the SA-responsive promoter of the *PR1* gene (Cao et al., 1994; Mou et al., 2003), the constitutive *POLYUBIQUITIN 10* (*UBQ10*) gene promoter and the constitutive Cauliflower Mosaic Virus 35S RNA (*CaMV35S*) promoter, respectively. Both, *VENUS* and *mKATE2* were fused to the nuclear localization signal *N7* (Cutler et al., 2000), providing the nuclear-targeted reporter module *PR1-VENUS-N7* and the nuclear-targeted reference module *UBQ10-mKATE2-N7*. VENUS was selected as output reporter because of its fast and efficient maturation characteristics (Nagai et al., 2002), whereas mKATE2 was chosen as reference due to its highest brightness among the available and spectrally separable far-red fluorescent proteins (Piatkevich and Verkhusha, 2011). EGFP was tagged with the plasma membrane (PM) protein low temperature induced protein 6b (LTI6b), supplying the PM marker module *CaMV35S-EGFP-LTI6b* (Cutler et al., 2000). CLSM of transgenic plants harboring the modular COLORFUL-PR1pro construct should thus allow detection of contours, sizes, shapes, identities, and viability of individual cells, their relative position in a tissue context and the simultaneous quantification of reporter and reference signal outputs in their corresponding nuclei. We generated 30 independent BASTA-resistant transgenic lines in the Col-0 background and analyzed leaves of seedlings by CLSM after treatment with either mock or 0.5 mM sodium salicylate solutions for 24 h. Top-view maximum projections of CLSM z-stack images showed for all but one of these transgenic lines robust and constitutive expression of the reference and cell boundary markers as well as an inducible expression of the SA-responsive reporter (Figure 1B). Notably, corresponding side-view 3D projections (Figure 1C) and fluorescence intensity vs. depth correlation analyses (Figures 1D,E) revealed that sodium salicylate treatment induced high levels of reporter fluorescence in mesophyll cells, whereas the overlying epidermal cells responded heterogeneously. In particular, nuclei of the surface-exposed stomatal guard cells appeared to show significantly lower levels of SA-inducible VENUS fluorescence. In marked contrast, mKATE2 reference signals exhibited considerably lower intensities in cells of the deeper mesophyll layer, which was expected and can be explained by attenuation of fluorescence signals in deeper tissues due to light scattering, absorbance and refraction (Ntziachristos, 2010). In light of this, the reciprocal pattern observed for the reporter suggested substantial differences of its activity in epidermal and mesophyll cells. Reinforcing the idea of distinct tissue-specific signaling outputs, treatment with 0.5 mM sodium salicylate affected the intensities of the cell boundary marker particularly in mesophyll cells (Figure 1B), potentially reflecting cell type-specific induction of PM protein turnover, endocytosis or toxic effects.

To facilitate comparative large-scale data analyses on a quantitative and cell-specific level, we developed an ImageJ plugin called COLORFUL SPOTTER, which allows automated nuclear fluorescence detection and quantification in z-stack images (Figures 1F,G and Supplementary Figures S1A,B). For this, complex z-stacks are split into upper epidermal (Figure 1F and Supplementary Figure S1A) and lower mesophyll cell layer projections (Figure 1G and Supplementary Figure S1B). Subsequent processing by the software assigns cell-specific nuclear index numbers and quantifies individual fluorescence intensities (Figures 1H,I and Supplementary Figures S1C,D). Membrane marker-supported visual inspection then allows for classification of epidermis cells into pavement/stomatal subpopulations and manual data curation. Curation is required when close nuclear signals are merged into a single spot (signals #34 and #39 in Figure 1F and signal #36 in Supplementary Figure S1A), when mesophyll-specific signals are erroneously detected in the epidermis layer (or vice versa) due to leaf curvature (signal #19 in Supplementary Figure S1A), or when merged and misassigned signals occur in combination (signal #40 in Supplementary Figure S1B). Consequently, every single z-stack image subjected to the described procedure provides a double-digit number of individual cell type-specific quantitative reporter and reference data points (Figures 1H,I and Supplementary Figures S1C,D), which are averaged to provide a single cell-type specific replicate. Standardized replicate experiments thus provide statistically robust data sets for guard, pavement and mesophyll cell-specific response studies (Figure 1J, Supplementary Figure S1E, and following figures). Indeed, our quantitative analyses clearly confirmed distinct cell-specific reporter activities, with epidermal guard cells being unresponsive to SA treatment, whereas epidermal pavement and palisade mesophyll cells showed a 4.5-fold (±0.73 SEM) and a 31-fold (±4.80 SEM) induced reporter activity, respectively (Figure 1J).

In principle, our system allows ratiometric hormone/reference reporter analyses. However, we noted that SA treatment had slight, but significant effects on the *UBQ10* promoter-driven mKATE2 reference in guard [1.15-fold (±0.04 SEM)] and mesophyll cells [1.5-fold (±0.19 SEM.)] (Supplementary Figure S1E). Thus, we decided not to conduct ratiometric fluorescence measurements in this study and to utilize mKATE2 reference fluorescence only for general nuclear detection, sorting and as a cellular viability reporter. Next, we tested hormone responsiveness of four independent transgenic SA reporter lines in epidermal pavement cells and found significantly similar quantitative readouts (Supplementary Figure S1F). For all subsequent experiments we used COLORFUL-PR1pro reporter line #1 and its filial generations obtained by self-fertilization or crossing with indicated mutant lines. Stable expression of all three reporter cassettes was observed at least until generation T4 (data not shown). Experiments conducted with homozygous *npr1*-1 SA signaling mutant plants (Cao et al., 1994) generated via crossing showed no inducible hormone reporter activity (Figure 1J), demonstrating functionality and specificity of our reporter. In contrast, dose response assays performed with our selected wildtype standard line showed significant increase in reporter activity upon treatment with 50 μm SA and optimal inducibility with 0.5 mM SA (Supplementary Figure S1G). Treatment of seedlings with 1 mM hormone solutions resulted in drastic reduction of plasma membrane marker fluorescence and tissue viability (data not shown), indicative of a critical threshold for cell death execution in *A. thaliana* seedlings grown under local standard growth conditions. Consequently, we set 0.5 mM sodium salicylate as the standard concentration in all experiments. Next, we analyzed the kinetics of the SA stimulus-dependent hormone reporter in epidermal pavement cells and observed statistically significant induction of activity at 6 h post treatment (hpt) and a gradual increase of VENUS fluorescence within the next 18 h (Supplementary Figure S1H). Finally, we compared the gradual increase in reporter activities with transcriptional activation of the *PR1* marker gene itself. To this end, the expression levels of *PR1* were quantified by classical qRT-PCR using using whole-leaf RNA preparations of wildtype Col-0 plants after treatments with 0.5 mM SA at 1.0, 3.0, 6.0, 12, and 24 hpt (Supplementary Figure S2A). These experiments confirmed the well-known SA-inducible expression of *PR1* in our experimental set-up and correlated well with the activity kinetics of our reporter (Supplementary Figure S1H).

### COLORFUL-VSP2pro and COLORFUL-PDF1.2apro Reporters Allow Hormonal Cross-Talk Analyses

**FIGURE 2:**
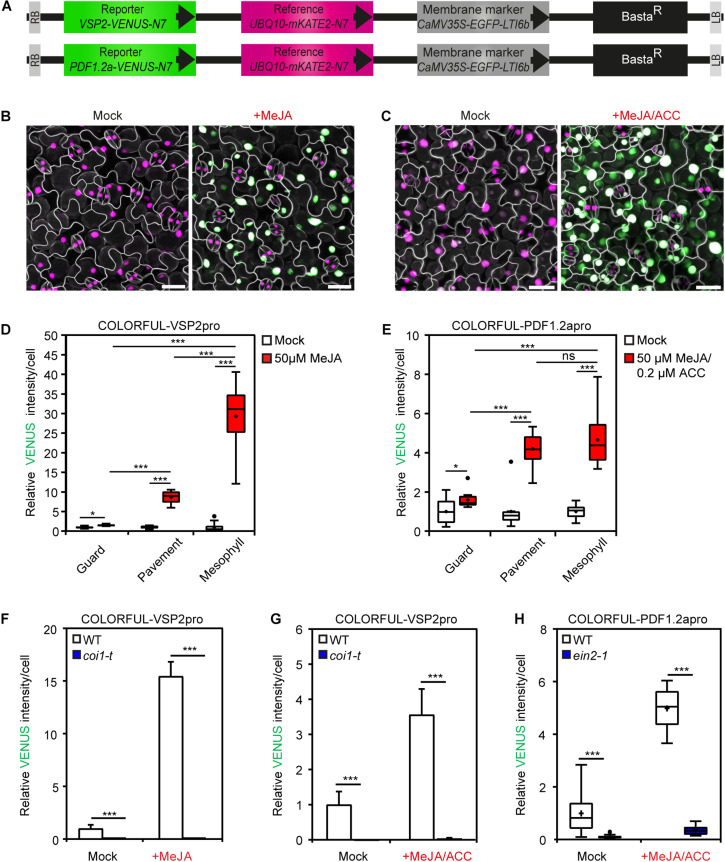
COLORFUL-VSP2pro and -PDF1.2apro reporter lines allow spatial mapping of JA and JA/ET signaling outputs. **(A)** Schematic representation of T-DNA construct harboring the fluorescent modules of the COLORFUL-VSP2pro (top) and COLORFUL-PDF1.2apro (bottom) reporters. Basta^*R*^, selectable marker; LB/RB, left/right borders of T-DNA. **(B,C)** Maximum projection of CLSM z-stack images showing overlays of VENUS (green), mKATE2 (magenta) and EGFP (gray) reporter expression in leaves of 12-day-old transgenic *A. thaliana* lines COLORFUL-VSP2pro#1 **(B)**, and COLORFUL-PDF1.2apro#1 **(C)** at 24 h post incubation with mock (left) or hormone solutions (right). Scale bar, 25 μm. **(D,E)** COLORFUL-VSP2pro **(D)** and -PDF1.2apro **(E)** reporter activities in leaf guard, pavement and palisade mesophyll cells of transgenic lines COLORFUL-VSP2pro#1 and COLORFUL-PDF1.2apro#1, respectively. **(F)** JA reporter activities in leaf pavement cells of COLORFUL-VSP2pro#1 (WT) or JA signaling mutant *coi1*-t. **(G,H)** Reporter activities in leaf pavement cells of COLORFUL-PDF1.2apro#1 (WT), *coi1*-t **(G)** or ET signaling mutant *ein2-1* **(H)**. Eleven-day-old seedlings were incubated in 50 μm MeJA **(B,D,F)** or 50 μm MeJA in combination with 2 μm ACC **(C,E,G,H)** for 24 h. Box plots show first quartile (lower line); median (center line); mean (+); third quartile (upper line); whiskers extend 1.5 times the interquartile range, and outliers (dots), *n* = 10. Bar charts show means ± SEM, *n* = 3–5. Data are relative to mock of WT. Asterisks indicate significant differences (ns not significant, *p < 0.05, ***p < 0.001, one-tailed Student’s *t*-test; see also Supplementary Figures S2B,C, S3, S4B,C).

**FIGURE 3:**
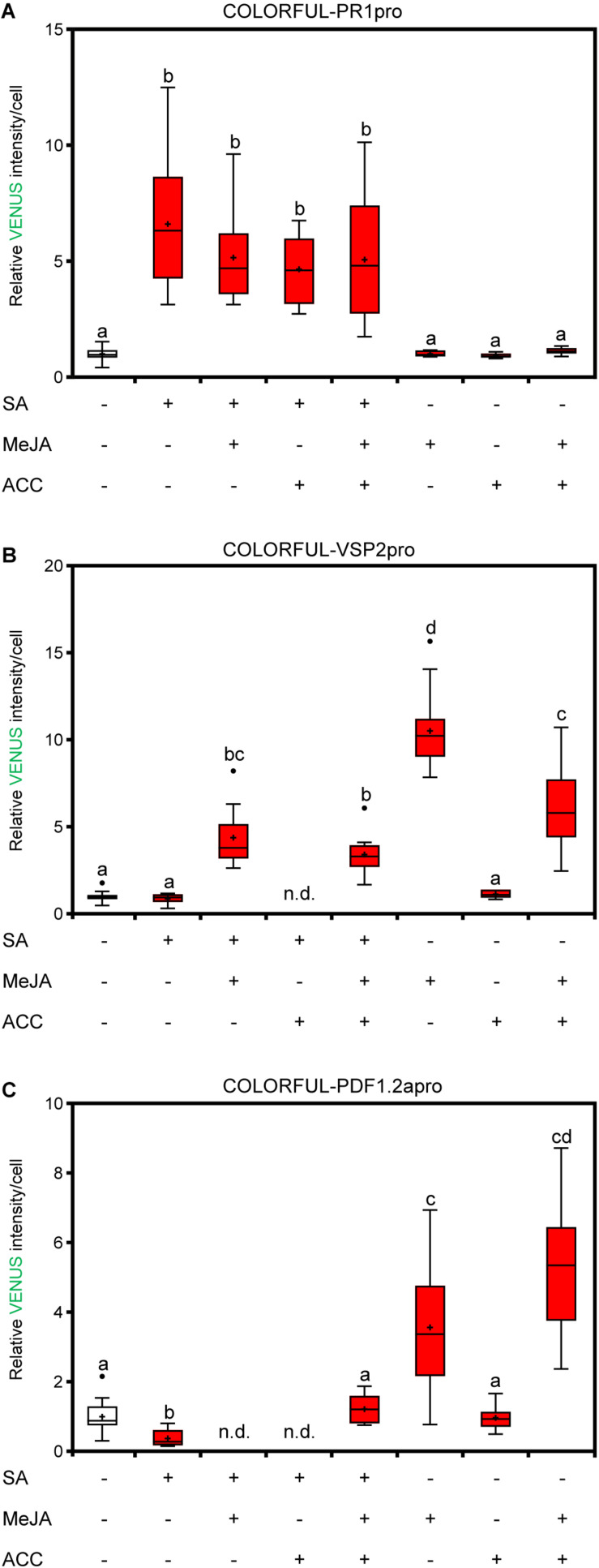
COLORFUL reporters reveal SA-JA-JA/ET crosstalk at the single-cell level. **(A–C)** COLORFUL-PR1pro **(A)**, COLORFUL-VSP2pro **(B)**, and COLORFUL-PDF1.2apro **(C)** reporter activities in leaf pavement cells of 12-day-old *A. thaliana* lines COLORFUL-PR1pro#1, -VSP2pro#1, and -PDF1.2apro#1, respectively, at 24 h post treatment with 0.5 mM sodium salicylate, 50 μm MeJA, 2 μm ACC or combined solutions. Box plots show first quartile (lower line); median (center line); mean (+); third quartile (upper line); whiskers extend 1.5 times the interquartile range, and outliers (dots), *n* = 8–15. Data are relative to the mean values of the corresponding mock. Different letters indicate significant differences (one-way ANOVA followed by Tukey’s multiple comparison test, *p* < 0.05).

To analyze the cell-specific activities of JA and JA/ET signaling pathways, we generated analogous COLORFUL-VSP2pro and COLORFUL-PDF1.2apro reporter constructs utilizing the JA-inducible promoter of the *VSP2* gene (Anderson et al., 2004; Lorenzo et al., 2004) and the JA/ET-responsive promoter of the *PDF1.2a* gene (Penninckx et al., 1998), respectively (Figure 2A). Agrobacterium-mediated transformation of Col-0 plants provided 30 independent BASTA-resistant transgenic lines for each construct, which all showed constitutive reference and membrane marker expression. Treatment with 50 μm methyl jasmonate (MeJA) alone, or in combination with 2 μm of the ethylene precursor 1-aminocyclopropane-1-carboxylic acid (ACC) for 24 h also induced detectable activities of the two reporters (Figures 2B,C). MeJA- and MeJA/ET-inducible VENUS reporter activity measurements were then conducted with four independent transgenic COLORFUL-VSP2pro and–PDF1.2apro lines, respectively, which showed significantly similar quantitative responses (Supplementary Figures S3A,B). We selected COLORFUL-VSP2pro line #1 and –PDF1.2apro line #1 for all following experiments. COLORFUL SPOTTER-supported quantitative analyses again demonstrated cell type-specific differences of reporter activities, with epidermal guard cells being hardly responsive to MeJA or MeJA/ET treatment, whereas epidermal pavement cells showed 8.7-fold (± 0.47 SEM) and 4.2-fold (± 0.28 SEM) induced COLORFUL-VSP2pro and–PDF1.2apro reporter activities, respectively (Figures 2D,E). Interestingly, mesophyll cells showed pavement cell-like COLORFUL–PDF1.2apro reporter responses [4.7-fold (±0.44 SEM)], but very high COLORFUL-VSP2pro reporter activities upon MeJA treatment [29.4-fold (±2.66 SEM)] (Figures 2D,E), suggesting quantitatively different induction signatures of the individual COLORFUL reporters at the tissue level. Again, we noted slight, but significant hormone treatment-dependent effects on mKATE2 reference activities (Supplementary Figures S3C,D), supporting our decision to refrain from ratiometric analyses in this study. In order to demonstrate specificity of our COLORFUL-VSP2pro and –PDF1.2apro reporters, we isolated homozygous JA-insensitive *coi1-*t mutants (Mosblech et al., 2011) and ethylene-insensitive *ein2*-1 mutants (Guzman and Ecker, 1990) after crossing with our selected standard lines, which showed drastically reduced levels of inducible VENUS fluorescence upon treatment with MeJA or MeJA/ACC, respectively (Figures 2F–H). Experiments with variable hormone concentrations and time-course analyses confirmed dosage-dependency and tractable induction kinetics of both COLORFUL reporters (Supplementary Figures S3E–H). Again, the observed induction kinetics correlated with the well-known overall hormone-inducibility of endogenous *VSP2* and *PDF1.2a* expression in whole leave extracts of Col-0 plants that we analyzed via qRT-PCR (Supplementary Figures S2B,C). Next, we used hormone combination treatments to demonstrate the suitability of all three COLORFUL reporters for hormone crosstalk studies at cellular resolution (Figures 3A–C). These analyses underpinned the robust and SA-specific activity of the *PR1* promoter (Leon-Reyes et al., 2009; Figure 3A), the antagonistic effects of SA and ACC on *VSP2* promoter activity (Lorenzo et al., 2004; Leon-Reyes et al., 2009; Figure 3B), as well as the synergistic capacity of MeJA and ACC on *PDF1.2* promoter inducibility, which is antagonized by SA (Koornneef et al., 2008; Figure 3C). Finally, the three COLORFUL-biosensors also showed organ-specific reporter activities in *A. thaliana* leaves, cotyledons and roots (Supplementary Figures S4A–C), highlighting the capability of our reporters to map hormone signaling responses in distinct plant organs.

### COLORFUL Reporters Unveil Distinct Invasion Dynamics of Virulent and Avirulent *Hpa* Isolates

**FIGURE 4:**
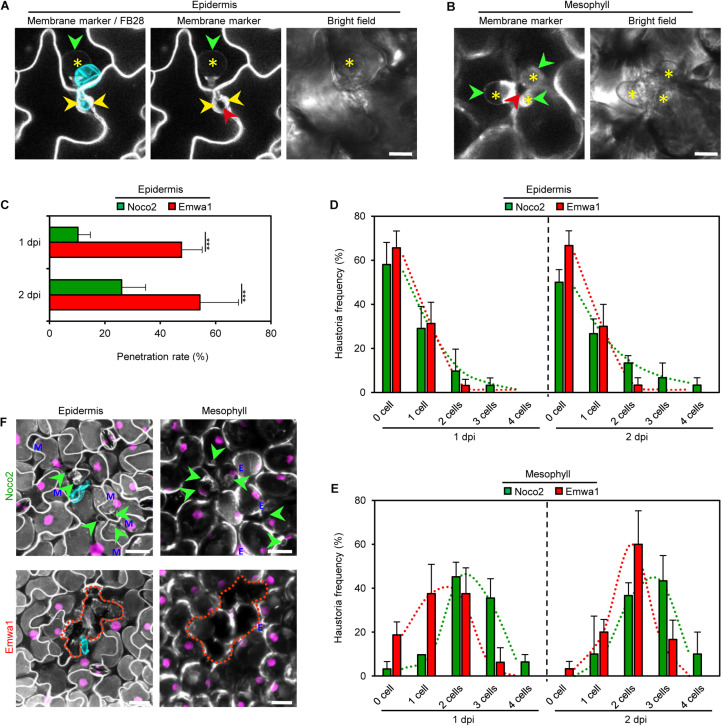
Virulent (Noco2) and avirulent (Emwa1) isolates of *H. arabidopsidis* exhibit distinct invasion dynamics on *A. thaliana* Col-0 leaves. **(A,B)** Maximum projections of CLSM z-stack images showing plasma membrane (PM) marker (gray; A, left and middle panels; B, left panel) in a leaf of a 3-week-old transgenic *A. thaliana* line COLORFUL-PR1pro#1 at 1 day post inoculation (dpi) with Emwa1 spore (cyan overlay; A, left) and after staining with Fluorescent Brightener 28 (FB28). PM allows visualization of penetration between outer periclinal epidermal cell walls (yellow arrowhead) by Emwa1, intercellular growth into anticlinal cell walls (red arrowheads; **A**, middle; **B**, left), and extrahaustorial membrane (green arrowheads), and haustoria formation (asterisks) in pavement cell **(A)** and palisade mesophyll cells **(B)**. Bright field images (right) show single plane sections. Scale bar, 10 μm. See also Videos S1 and S2. **(C)** Noco2 and Emwa1 penetration rates in leaves of 3-week-old COLORFUL lines. Penetration was determined as growth into anticlinal cell walls between epidermal cells (red arrowhead; **A**) at 1 and 2 day(s) post inoculation (dpi). **(D,E)** Frequency of haustoria establishment by Noco2 and Emwa1 determined as formation of extrahaustorial membrane (green arrowheads; **A,B**) in epidermal **(D)** and mesophyll cells **(E)** at 1 and 2 dpi. Measurements were obtained from three independent experiments, each containing 10 biological replicates. Data represent means ± SEM **(F)** Maximum projection of CLSM z-stack images showing overlays of reference (magenta) and PM (gray) markers in leaves of 3-week-old *A. thaliana* line COLORFUL-PR1pro#1 at 3 dpi after staining Noco2 (top) and Emwa1 (bottom) spores with FB28 (cyan). While Noco2 growth indicated by haustoria formation (green arrowheads) was extended in epidermis (left) and mesophyll (right) beyond the initial site of invasion, Emwa1 induced cell death (discontinuous lines) associated with disappearance of PM and reference fluorescence. Blue E and M indicate reference signals originating from epidermal and mesophyll cells. Scale bar, 25 μm (see also Supplementary Videos S3,S4 and Supplementary Figure S6A).

Next, we used our three COLORFUL reporter lines for comparative analyses of spatiotemporal hormone alterations and crosstalk in compatible and incompatible interactions of *A. thaliana* with different isolates of the oomycete pathogen *Hpa*. Notably, the constitutively expressed PM marker EGFP-LTI6b turned out to be ideally suited to monitor individual plant-microbe interaction sites, penetration of outer periclinal epidermal cell walls, intercellular growth into anticlinal epidermal cell walls and invasive establishment of haustoria in epidermal and mesophyll cells (Figures 4A,B and Supplementary Videos S1, S2). Our analyses showed that both, the virulent isolate Noco2 and the avirulent isolate Emwa1 penetrate, grow intramurally and establish haustoria in epidermal and mesophyll cells of the *A. thaliana* ecotype Col-0, albeit at different rates (Figures 4C–E), suggesting distinct isolate-specific infection kinetics and invasion success. Interestingly, initial penetration and intramural growth occurred at considerably higher levels in inoculation experiments with avirulent Emwa1 (Figure 4C). However, the enhanced invasiveness and virulence of Noco2 was clearly reflected by higher frequencies of haustoriated epidermal, and in particular, mesophyll cells at 1 dpi, and, even more pronounced, at 2 dpi (Figures 4D,E). At 3 dpi, HR-like cell death responses, indicated by loss of PM and nuclear reference marker fluorescence, characterized individual sites of interaction between Emwa1 and Col-0 epidermis and mesophyll cells (Figure 4F, lower panel and Supplementary Video S3). As expected, in compatible interactions with Noco2 presence of haustorial complexes correlated with maintained plant tissue integrity reflected by unaltered PM and nuclear reference marker fluorescence (Figure 4F, upper panel; Supplementary Video S4).

### *A. thaliana-Hpa* Interaction Sites Show Spatio-Temporally Distinct SA, JA, and JA/ET Signaling Signatures

**FIGURE 5:**
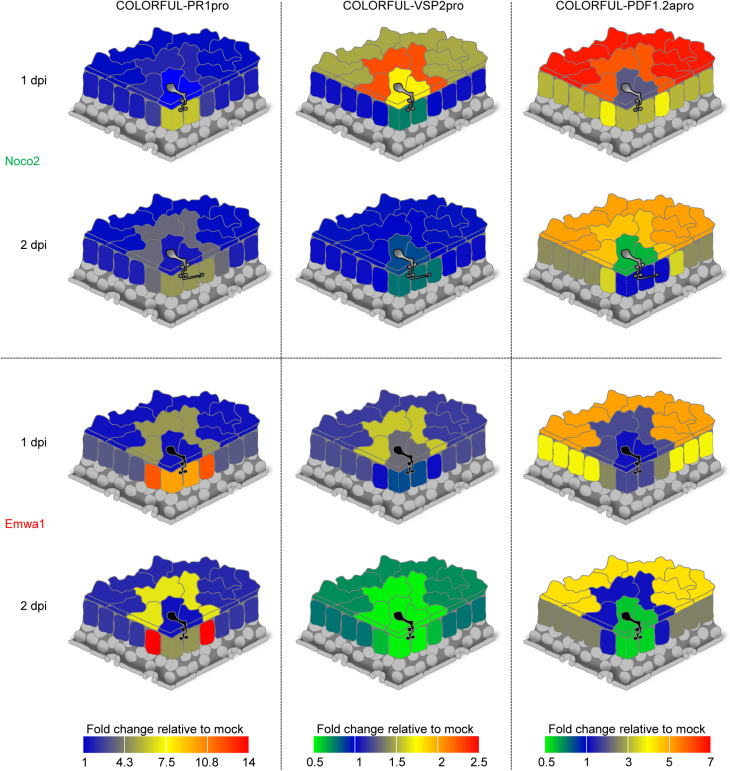
Spatio-temporal signatures of SA, JA, and JA/ET signaling activities at sites of *A. thaliana-H. arabidopsidis* interactions. Quantitative heat map representations of COLORFUL-PR1pro (left), COLORFUL-VSP2pro (middle), and COLORFUL-PDF1.2apro (right) reporter activities in leaves of 3-week-old *A. thaliana* lines COLORFUL-PR1pro#1, -VSP2pro#1 and -PDF1.2apro#1, respectively, at 1 (top) and 2 (bottom) day(s) post inoculation (dpi) with virulent (Noco2; uppermost two schemes) and avirulent (Emwa1; lowermost two schemes) isolates of *H. arabidopsidis* (see also Supplementary Figure S7).

In agreement with earlier results obtained in other labs (Mauch-Mani and Slusarenko, 1996), UPLC-nano ESI-MS/MS-based hormone measurements and qRT-PCR analyses conducted with whole leaf samples showed that resistance against Emwa1 mediated by the Resistance (R) gene product RESISTANCE TO PERONOSPORA PARASITICA 4 (RPP4) (van der Biezen et al., 2002) is accompanied by significant accumulation of free SA and concomitantly triggered *PR1* gene expression (Supplementary Figures S5A,D). Free JA and jasmonoyl-l-isoleucine (JA-Ile) quantification as well as *VSP2* gene expression analyses confirmed unaltered levels in both Noco2- and Emwa1-challenged leaf samples harvested at 1 and 2 dpi (Supplementary Figures S5B,C,E), whereas significantly induced *PDF1.2* transcription was observed in Col-0 whole leaf tissue material at 2 dpi with both virulent Noco2 and avirulent Emwa1 (Supplementary Figure S5F). However, systematic COLORFUL reporter line analyses using microscopic fields of view containing a single oomycete-plant interaction site revealed distinct reporter activity signatures in epidermis and mesophyll cells depending on the presence of and distance to oomycete infection structures (see below). Consequently, we categorized epidermal and mesophyll cells into “invaded,” immediately “adjacent,” and “distant” cells (Supplementary Figures S6A,B), and conducted quantitative COLORFUL-PR1pro,-VSP2pro, and –PDF1.2apro reporter analyses at 1 and 2 dpi with Noco2 or Emwa1 (Figure 5 and Supplementary Figure S7).

Our studies revealed that in contrast to haustoriated mesophyll cells, in which both *Hpa* isolates induced high levels of COLORFUL-PR1pro reporter fluorescence at 1 dpi, epidermal pavement cells did not show any altered SA reporter activity in response to invasion by either Noco2 or Emwa1 (Figure 5, left panel and Supplementary Figures S7A,B). Interestingly, however, in incompatible interactions with avirulent Emwa1, the highest levels of COLORFUL-PR1pro reporter induction were observed in the immediately adjacent mesophyll cells at 1 dpi (13.0-fold), and at 2 dpi (13.8-fold). A similar activation signature was detectable in pavement cells adjacent to Emwa1-invaded epidermal cells at 1 and 2 dpi, but at lower levels (4.7- and 7.0-fold, respectively). Together, these data suggest a major role of mesophyll and pavement cells immediately adjacent to sites of direct plant-microbe interaction in SA-dependent defense against avirulent Emwa1. Comparatively subtle induction of the COLORFUL-PR1pro reporter was also detectable in pavement and mesophyll cells neighboring Noco2-invaded cells at 2 dpi (3.5- and 3.1-fold, respectively), indicative of a conserved and potentially suppressed SA-controlled basal defense signature, which is reinforced in RPP4-dependent immunity triggered by Emwa1 (Figure 5 left panel and Supplementary Figures S7A,B).

Parallel COLORFUL-VSP2pro reporter analyses showed transiently enhanced activities in invaded and adjacent pavement cells at 1 dpi with Noco2 (1.8- and 2.2-fold, respectively), whereas Emwa1 triggered a similar response only in adjacent pavement cells (1.6-fold) (Figure 5, middle panel and Supplementary Figure S7C). Later, at 2 dpi, Emwa1 interaction sites were characterized by suppressed overall JA activity in pavement and mesophyll cells, which is particularly prominent in adjacent mesophyll cells (0.6-fold) (Figure 5, middle panel and Supplementary Figure S7D). Together, these data provide distinct cell type-specific JA response signatures for compatible and incompatible *Hpa* interactions that likely reflect antagonistic intra- and intercellular crosstalk with the SA pathway.

COLORFUL-PDF1.2apro reporter studies revealed 2.3-fold induced activity in Noco2-invaded pavement cells and remarkably high signals in immediately adjacent and distant epidermal pavement cells at 1 dpi (6.1- and 6.7-fold, respectively) (Figure 5, right panel and Supplementary Figure S7E), suggesting JA/ET-dependent rapid and gradual long-distance intercellular basal defense signaling in the epidermal tissue. At 2 dpi, Noco2-invaded pavement cells showed repressed COLORFUL-PDF1.2apro reporter activity (0.7-fold), whilst adjacent and distant pavement cells still had elevated, but lower marker fluorescence than at 1 dpi (4.7- and 4.9-fold, respectively) (Figure 5, right panel and Supplementary Figure S7F). Similarly, long-distance signaling was also detectable in the mesophyll tissue (3.1-fold at 1 dpi and 2.7-fold at 2 dpi, respectively), whereas highest activities of the COLORFUL-PDF1.2apro reporter occurred in immediately adjacent cells (3.9-fold at 1 dpi and 3.3-fold at 2 dpi, respectively). Emwa1-inoculated plants showed similar responses in distant tissues. However, in contrast to Noco2, Emwa1 did not trigger JA/ET signaling in the adjacent cell zone, suggesting a potential antagonistic crosstalk with the SA pathway. At 2 dpi, Emwa1-invaded epidermal pavement and mesophyll cells destined to undergo HR-like cell death within the next 24 h (Figure 4F), displayed attenuated COLORFUL-PDF1.2apro reporter activity. Notably, immediately adjacent cells exhibited the highest relative fold changes observed for the COLORFUL-PR1pro reporter in this study, possibly representing a demarcation signature controlling initiation and local containment of the cell death response in ETI.

## Discussion

In this work, we established transgenic *A. thaliana* reporter lines that enabled mapping of SA, JA and JA/ET signaling outputs at single-cell resolution. In contrast to previously reported *PR1*, *PDF1.2a* and *VSP2* promoter-based reporters (Manners et al., 1998; Murray et al., 2002; Mousavi et al., 2013; Betsuyaku et al., 2017; Poncini et al., 2017; Marhavy et al., 2019), our COLORFUL biosensors feature a reference readout and a cell membrane marker module. The reference reporter allows detection of all nuclei in the microscopic field of view, thus including nuclei without detectable hormone reporter activity, which would potentially be missed in set-ups using a hormone-controlled reporter only. Moreover, it serves as a cellular viability marker, allows robust and precise quantification of single-cell reporter activities in distinct cell types and can optionally be used for ratiometric reporter/reference analyses. Another key feature of our COLORFUL system is a cell membrane marker module that we used to allocate the single-cell signals and to monitor tissue integrity, pathogen invasion and induced cell death responses. Moreover, we automatized the quantification of single-cell fluorescence signals with a COLORFUL SPOTTER plugin tool, which supports high-throughput confocal image processing and cellular hormone response analyses at every research institution equipped with a standard confocal microscope.

Here, we focused on plant leaf tissues and found remarkably distinct cell-type-specific hormone sensitivities and signaling outputs. Palisade mesophyll cells beneath the upper epidermis showed highly responsive reporter activities upon exogenous SA and JA applications. This may explain why stealth pathogens like the obligate powdery mildew fungi evolved an ectoparasitic lifestyle, restrict invasion structures to epidermal pavement cells (Kuhn et al., 2016; Ma et al., 2016) and avoid deeper tissues with potentially stronger defense capacities. In marked contrast, surface-exposed stomatal guard cells showed hyposensitivity upon SA, JA, and JA/ET treatments. Given that mature guard cells are not connected to epidermal pavement and palisade mesophyll cells via plasmodesmata (Wille and Lucas, 1984) this may reflect reduced hormone uptake from the apoplastic space. Alternatively or additionally, cell type-specific expression of regulatory signaling components may also contribute to the lower reporter outputs that we generally observed in guard cells. This scenario is supported by previously described high guard cell-specific expression levels of the JA signaling repressor JASMONATE ZIM DOMAIN 2 (JAZ2), which prevents stomatal reopening and confers resistance to bacterial invasion. Conceivably, other SA, JA, and JA/ET signaling components may also show cell-type-specific activity patterns. In the future, reporters such as our COLORFUL biosensors may facilitate identification of novel cell-specific master regulators in mutant screens using automated high-throughput confocal imaging, uncover so far overlooked tissue-specific activities of already established signaling hubs, and thereby contribute to clarify seemingly contradictory or inconsistent findings.

Here, we utilized the COLORFUL reporter lines to trace the spatio-temporal dynamics of SA, JA and JA/ET signaling outputs at nascent interaction sites with virulent and avirulent *Hpa* isolates Noco2 and Emwa1 in *A. thaliana* leaf tissues. Our results demonstrated that standard crude leaf extract-based hormone measurements and qRT-PCR marker gene analyses have limited informational value as spatially restricted signaling and gene activities are averaged and thus lost. Similarly important for comparative analyses, but often neglected too, are distinct pathogen invasion kinetics as evidenced here for Noco2 and Emwa1, as they have considerable effects on timing and amplitude of plant defense responses.

By focusing on individual plant-microbe interaction sites, we were able to show here that both, virulent and avirulent isolates induce SA-dependent signaling outputs in haustoriated mesophyll and immediately adjacent cells. These alterations in SA signaling at the nascent site of attack are in line with the well-documented role of SA in basal defense against virulent *Hpa* isolates as well as RPP4-mediated resistance (Delaney et al., 1994; Mauch-Mani and Slusarenko, 1996; McDowell et al., 2000; van der Biezen et al., 2002; Asai et al., 2014). Notably, however, invaded pavement cells did not exhibit elevated COLORFUL-PR1pro reporter activities, whereas neighboring cells showed particularly high responsiveness in Emwa1 interactions. Previously, Caillaud et al. (2013) showed that virulent *Hpa* Waco9 at late time of tissue colonization (6 dpi) actively suppresses *PR1* promoter-driven GUS expression in attacked mesophyll cells. Suppression of PR1-dependent SA signaling was suggested to be mediated by the *Hpa* effector HaRxL62 (Asai et al., 2014), which we found to be conserved in Noco2 and Emwa1 (data not shown). Together, these results indicate that virulent and avirulent isolates efficiently suppress PR1-dependent SA signaling in invaded epidermal cells, but that hypersensitive mesophyll cells and in particular adjacent cells mount a strong SA output response in ETI. This prominent boost of COLORFUL-PR1pro reporter activity provides an ETI-specific spatial signature, which conspicuously correlates with the cellular boundaries of the ensuing HR-like cell death response. In support of a mechanistic context, SA is known to induce the expression of *PDLP5*, a plasmodesmatal protein that controls cell-to-cell communication and triggers cell death (Lee et al., 2011). Conceivably, a complex signaling circuit and well-coordinated intercellular communication are required for activation and local containment of cell death, which likely involves antagonistic hormone activities. In both, compatible and incompatible interactions, JA signaling outputs were only marginally altered. Possibly, this reflects a minor role of JA-dependent signaling in *A. thaliana-Hpa* interactions, which is supported by Thomma et al. (1998) who found no detectable changes of susceptibility to *Hpa* in JA treatment experiments and in the JA receptor mutant *coi1*. In marked contrast, we observed inverted output signatures of the COLORFUL-PDF1.2apro and COLORFUL-PR1pro reporters during incompatible Emwa1 interactions in adjacent and distant cells. This may suggest that antagonistic JA/ET signaling processes restrict the boost of the SA signal and cell death execution to the site of pathogen attack, thereby minimizing plant tissue damage and mitigating the growth-defense tradeoff in ETI (Karasov et al., 2017). JA/ET signaling was also, but at considerably higher levels, triggered in cells immediately adjacent and distant to sites of Noco2 invasion. This finding is consistent with the upregulation of *PDF1.2* expression in *A. thaliana* infected with the virulent *Hpa* isolate Waco9 (Caillaud et al., 2013), which is mediated by the *Hpa* effector HaRxL44 in order to neutralize the activity of the SA pathway and to enhance biotrophy.

Betsuyaku et al. (2017) recently reported spatially different zones of SA and JA activities around *A. thaliana* leaf infection sites infiltrated with *Pseudomonas syringae* pv. *tomato* DC3000 carrying AvrRpt2, using similar nuclei-targeted *PR1*- and VSP1-driven fluorescent reporters. However, the experimental set-up of this study, which lacked nuclear reference and cell shape markers, employed infiltration of bacterial suspensions and exclusively focused on an incompatible interaction, precluded comparative and accurate quantitative analyses of individual cellular plant-microbe interaction sites at single-cell resolution. Thus, we believe that our work represents a significant advance and demonstrates at cellular resolution and quantitative precision, that basal and R-gene mediated resistance of *A. thaliana* against *Hpa* are characterized by markedly distinct spatio-temporal signatures of antagonistic hormone activities around the initial site of invasion. Future experiments will need to address the cell type-specific contribution of antagonistically active regulatory master proteins, such as NPR1, 3, and 4 (Fu et al., 2012; Ding et al., 2018) to this phenomenon. Moreover, comparisons or even combinations with biosensors that directly monitor phytohormone concentrations such as JAS9-VENUS (Larrieu et al., 2015) should provide precise insights into the inputs and outputs of hormone-controlled signaling at the single cell level.

## Data Availability Statement

All datasets generated for this study are included in the article/Supplementary Material, further inquiries can be directed to the corresponding authors.

## Author Contributions

HG, IF, and VL conceived and designed the experiments. HG, ME-S, OT, KH, and CH performed the experiments. MP and HG developed the COLORFUL SPOTTER plugin. HG, ME-S, IF, and VL analyzed and discussed the data. HG and VL wrote the manuscript. All authors contributed to the article and approved the submitted version.

## Conflict of Interest

The authors declare that the research was conducted in the absence of any commercial or financial relationships that could be construed as a potential conflict of interest.

## Supplementary Material

Supplementary Figure 1 Functional characterization of *A. thaliana* COLORFUL-PR1pro reporter lines.

Supplementary Figure 2 Hormone-induced *PR1*, *VSP2*, and *PDF1.2a* gene expression monitored by qRT-PCR.

Supplementary Figure 3 Functional characterization of *A. thaliana* COLORFUL-VSP2pro and –PDF1.2apro reporter lines.

Supplementary Figure 4 COLORFUL-PR1pro, -VSP2pro, and –PDF1.2apro reporter activities in different *A. thaliana* organs.

Supplementary Figure 5 Virulent (Noco2) and avirulent (Emwa1) isolates of *H. arabidopsidis* induce distinct whole-leaf hormone accumulation and signaling output patterns.

Supplementary Figure 6 Dissection of cells associated with *H. arabidopsidis* (*Hpa*) invasion.

Supplementary Figure 7 SA, JA, and JA/ET signaling signatures at *A. thaliana-H. arabidopsidis* interaction sites.

Supplementary Method 1 Determination of Phytohormones by UPLC-nano ESI-MS/MS.

Supplementary Video 1 *H. arabidopsidis* isolate Emwa1 invading leaf epidermis of COLORFUL-PR1pro#1 at 1 day post inoculation. Site of penetration (yellow arrowhead), extrahaustorial membrane surrounding the haustoria (green arrowhead).

Supplementary Video 2 *H. arabidopsidis* isolate Emwa1 invading leaf palisade mesophyll of COLORFUL-PR1pro#1 at 1 day post inoculation. Extrahaustorial membrane surrounding the haustoria (green arrowhead).

Supplementary Video 3 *H. arabidopsidis* isolate Noco2 invading leaf of COLORFUL-PR1pro#1 at 3 days post inoculation. Extrahaustorial membrane surrounding the haustoria (green arrowhead).

Supplementary Video 4 *H. arabidopsidis* isolate Emwa1 invading leaf of COLORFUL-PR1pro#1 at 3 days post inoculation. Extrahaustorial membrane surrounding the haustoria (green arrowhead).
