# Supplementary figures and images for: Quantitative Hormone Signaling Output Analyses of Arabidopsis thaliana Interactions With Virulent and Avirulent Hyaloperonospora arabidopsidis Isolates at Single-Cell Resolution
Source: Front Plant Sci. 2020 Nov 6;11:603693. doi: 10.3389/fpls.2020.603693 (PMC7677359; doi:10.3389/fpls.2020.603693)

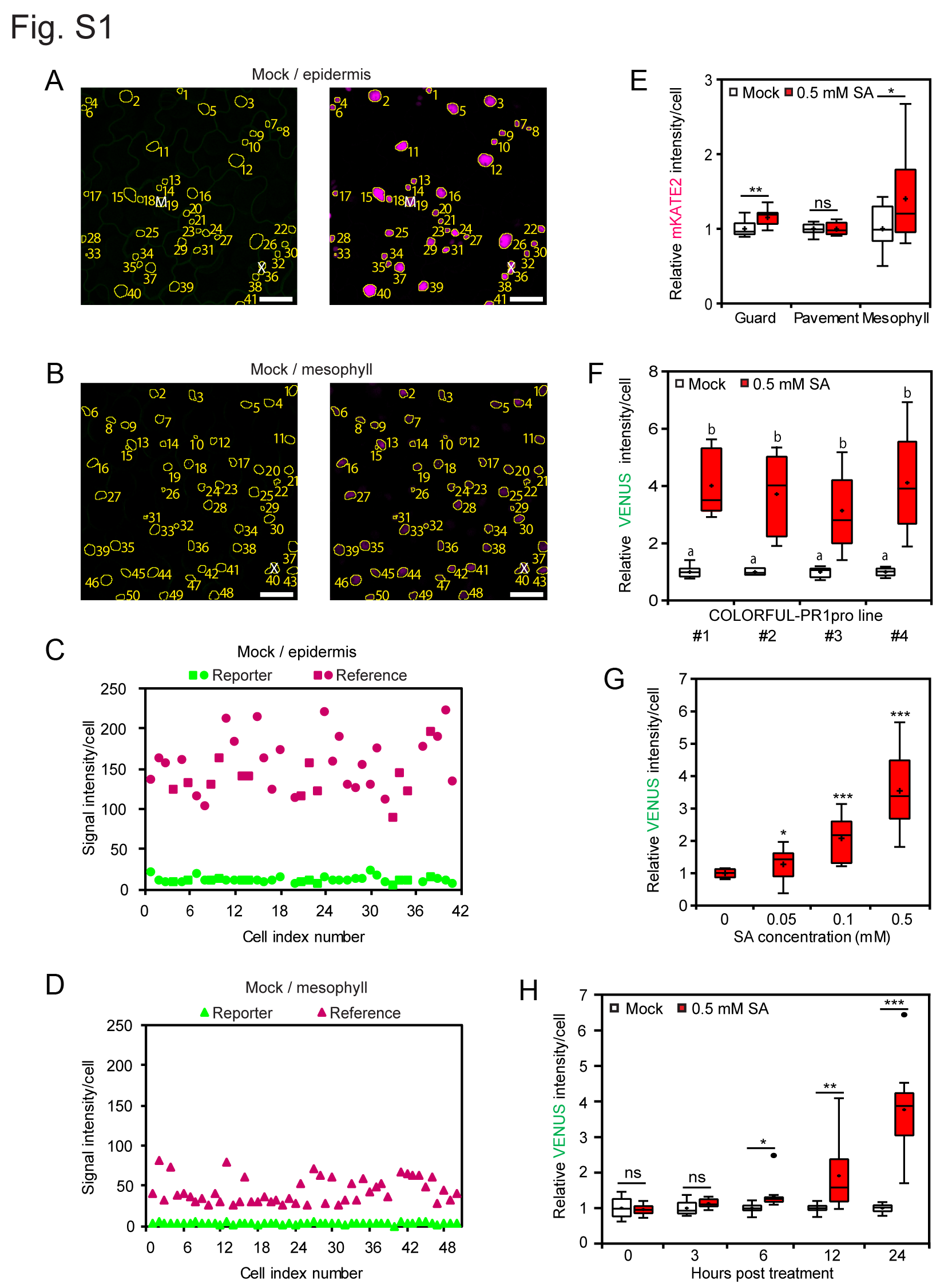

Supplement: Supplementary Figure 1 — Functional characterization of A. thaliana COLORFUL-PR1pro reporter lines. [file Image_1.TIF]

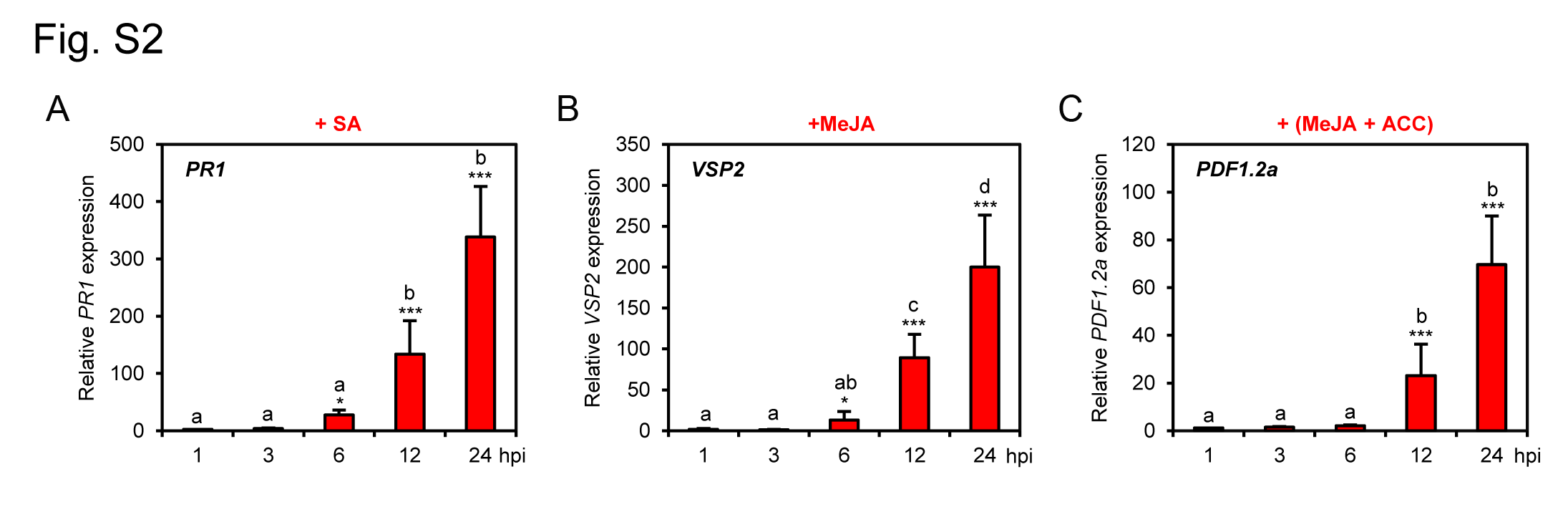

Supplement: Supplementary Figure 2 — Hormone-induced PR1, VSP2, and PDF1.2a gene expression monitored by qRT-PCR. [file Image_2.TIF]

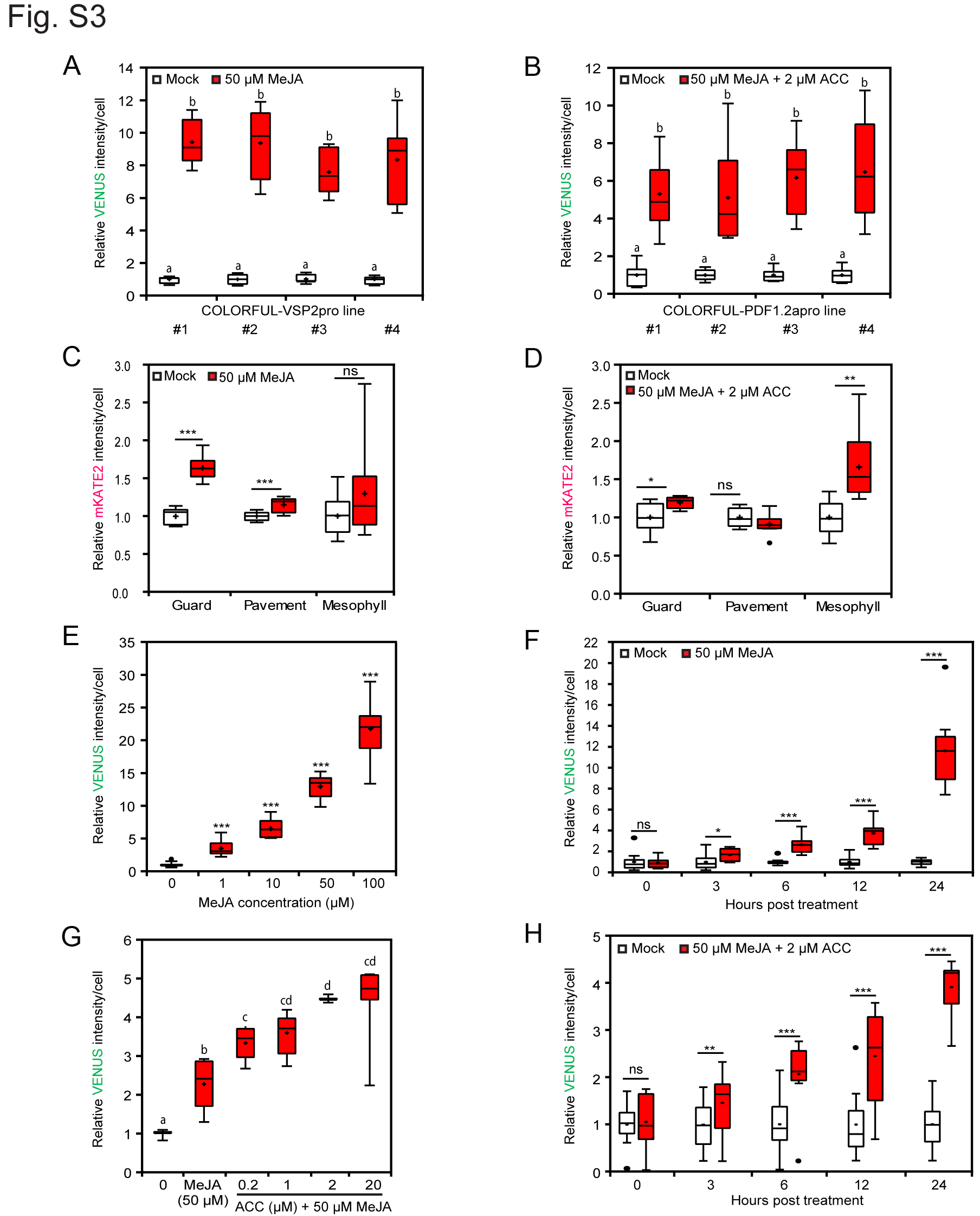

Supplement: Supplementary Figure 3 — Functional characterization of A. thaliana COLORFUL-VSP2pro and –PDF1.2apro reporter lines. [file Image_3.TIF]

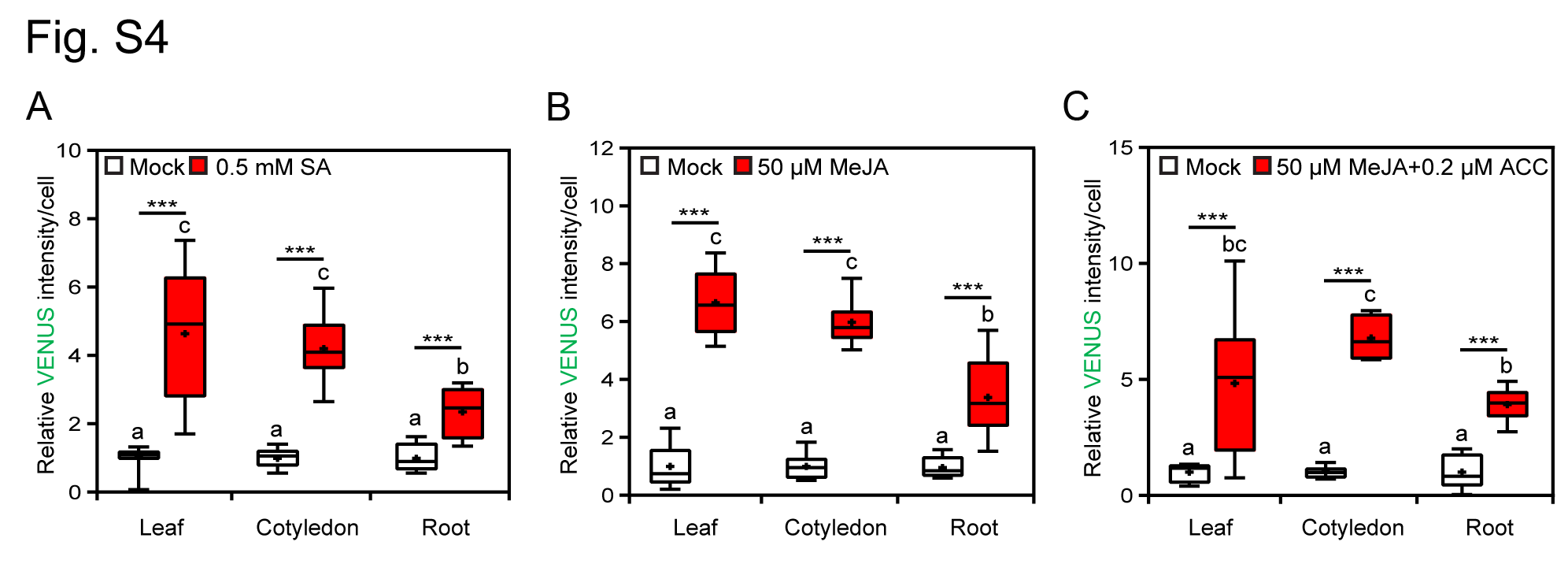

Supplement: Supplementary Figure 4 — COLORFUL-PR1pro, -VSP2pro, and –PDF1.2apro reporter activities in different A. thaliana organs. [file Image_4.TIF]

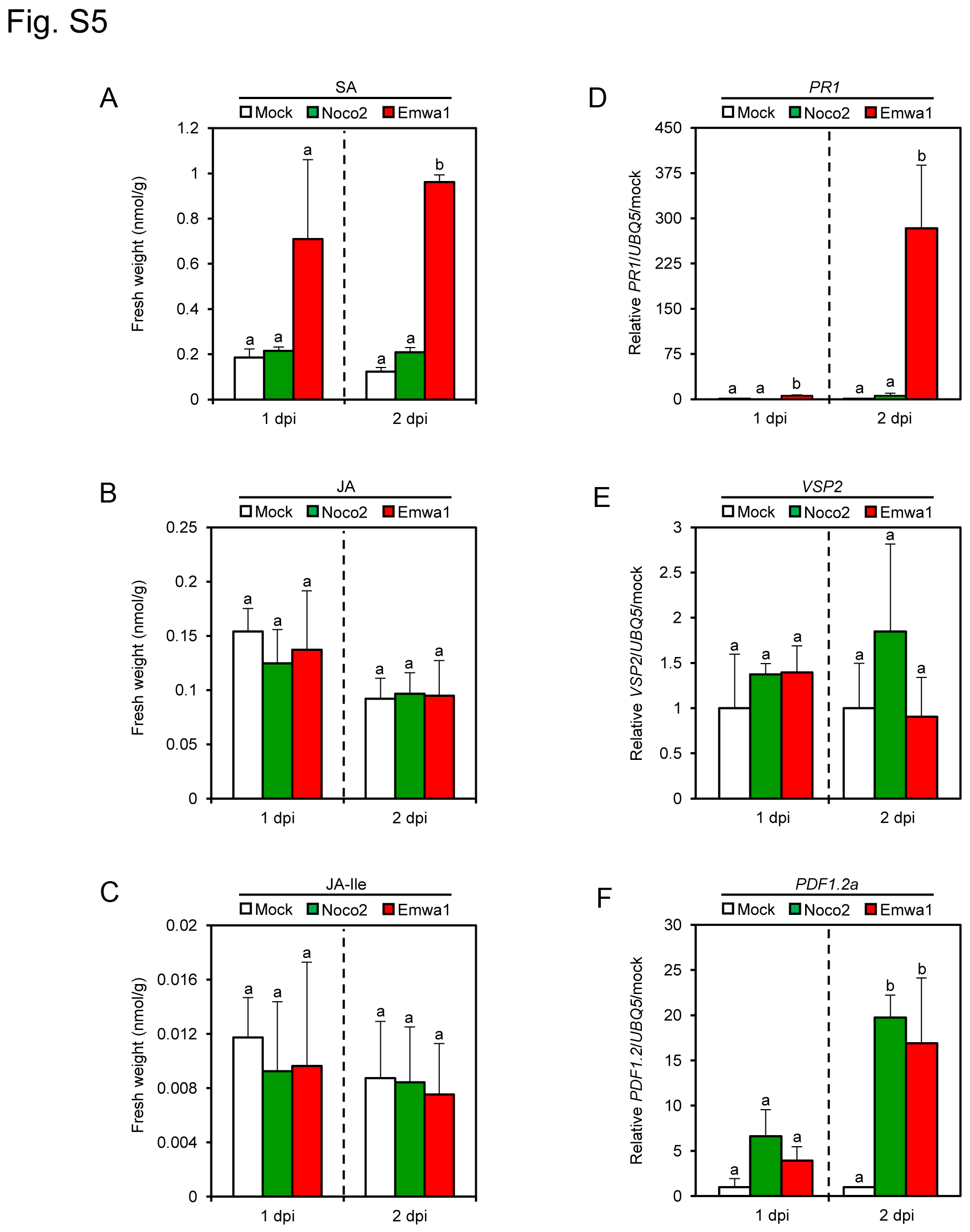

Supplement: Supplementary Figure 5 — Virulent (Noco2) and avirulent (Emwa1) isolates of H. arabidopsidis induce distinct whole-leaf hormone accumulation and signaling output patterns. [file Image_5.TIF]

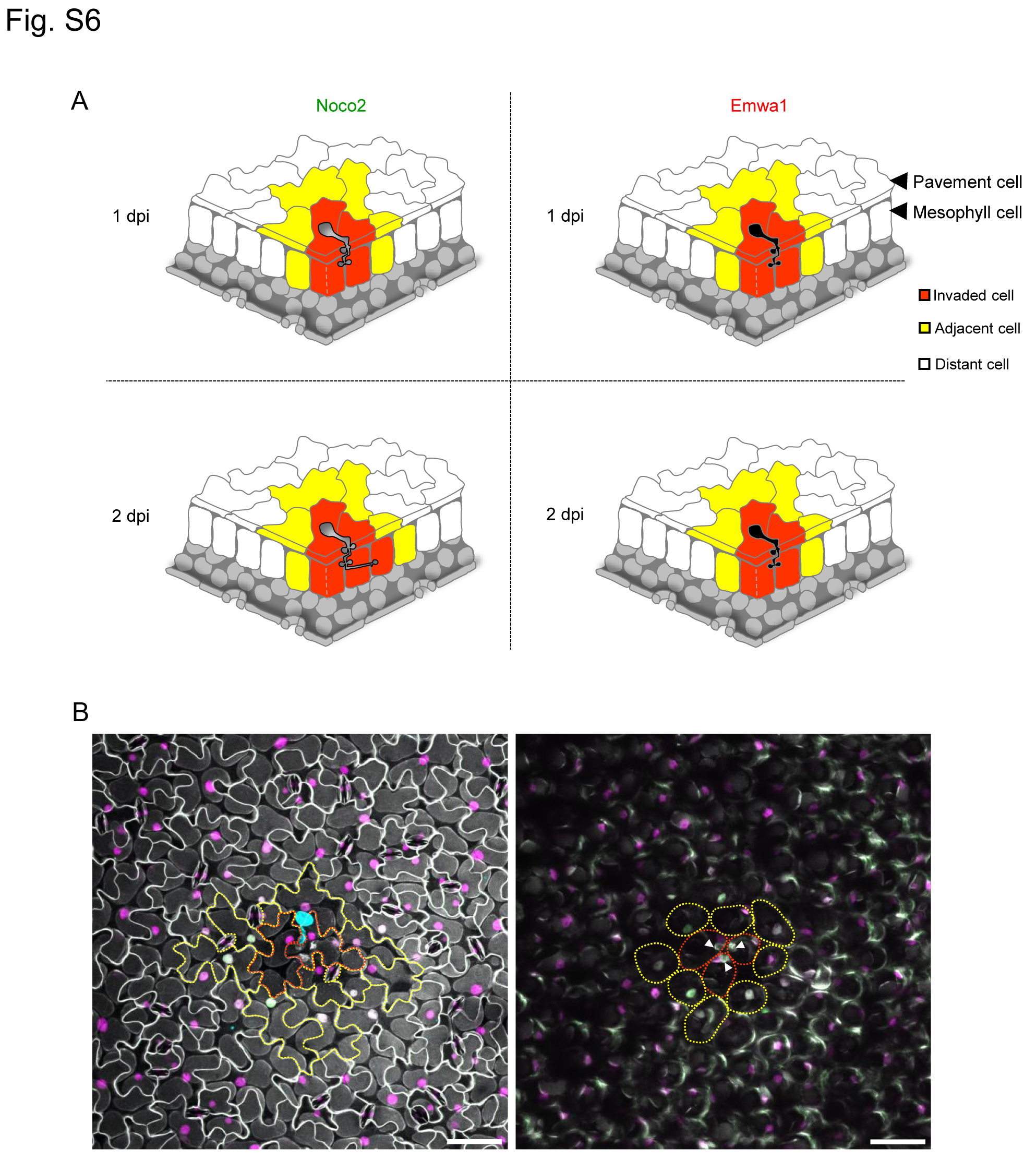

Supplement: Supplementary Figure 6 — Dissection of cells associated with H. arabidopsidis (Hpa) invasion. [file Image_6.TIF]

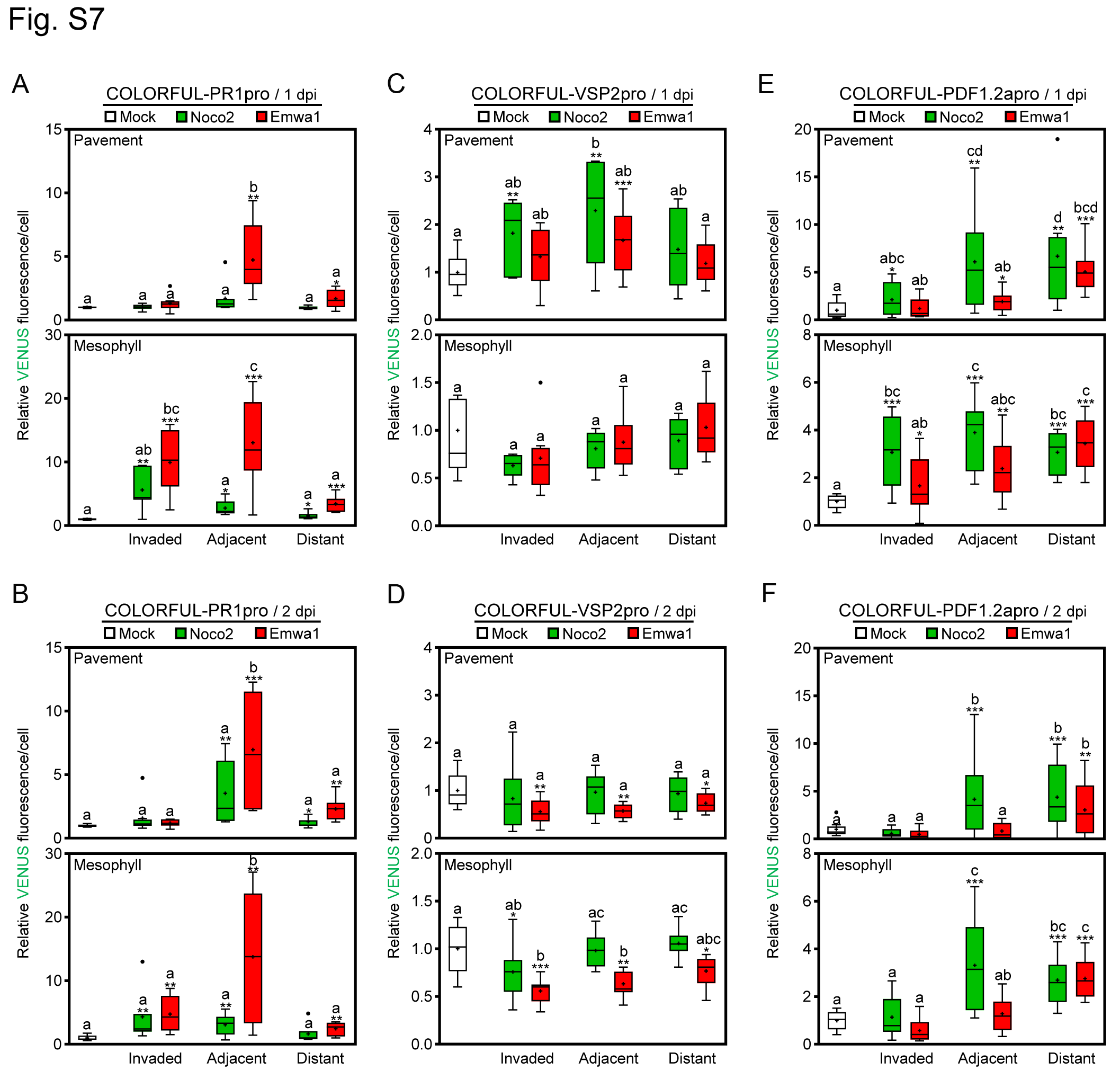

Supplement: Supplementary Figure 7 — SA, JA, and JA/ET signaling signatures at A. thaliana-H. arabidopsidis interaction sites. [file Image_7.TIF]
