# Supplementary material for: Quantitative Hormone Signaling Output Analyses of Arabidopsis thaliana Interactions With Virulent and Avirulent Hyaloperonospora arabidopsidis Isolates at Single-Cell Resolution
Source: Front Plant Sci. 2020 Nov 6;11:603693. doi: 10.3389/fpls.2020.603693 (PMC7677359; doi:10.3389/fpls.2020.603693)
Supplement: Supplementary file 8 [file Data_Sheet_1.DOCX]

**Supplementary Figure S1. Functional characterization of Arabidopsis COLORFUL-PR1pro reporter lines. (A,B) Detection of nuclear-localized fluorescence with COLORFUL SPOTTER in images of COLORFUL-PR1pro#1 leaf after mock treatment. Images represent merge of the fluorescence detection mask (yellow overlay) with maximum projections of z-stacks belonging to the epidermal (A) or mesophyll (B) cell layers. z-stacks were obtained from images presented in Figure 1b (top). Reporter (left) and reference (right). White X and M indicate manually curated spots resulting from erroneous merging of close nuclear signals into single spots or faulty tissue assignment of nuclear signals, respectively. Scale bar, 25 μm. (C, D) COLORFUL SPOTTER quantifications of single-cell-associated reporter and reference fluorescence signals from a and b, respectively. (E) Effect of sodium salicylate treatment on reference signal intensities in guard, pavement and palisade mesophyll cells of transgenic line COLORFUL-PR1pro#1. (F) Reporter activities in the leaf pavement cells of four independent COLORFUL-PR1pro transgenic lines. (G,H) Reporter activities in the leaf pavement cells of transgenic line COLORFUL-PR1pro#1 in response to dose (G) and duration (H) of treatment with sodium salicylate. Eleven-day-old seedlings were incubated in mock or 0.5 mM sodium salicylate solutions for 24 h, unless otherwise indicated.** The experiments were repeated twice showing consistent results. Box plots show first quartile (lower line); median (center line); mean (+); third quartile (upper line); whiskers extend 1.5 times the interquartile range, and outliers (dots), n = 9-10. Data show means ± s.e.m. Data are relative to the mean value of mock of WT. **Different letters indicate significant differences (Two-way ANOVA followed by Tukey’s multiple comparison test, p < 0.05). Asterisks indicate significant differences (ns not significant, *p < 0.05, **p < 0.01, ***p < 0.001, one-tailed Student’s t-test).**

**Supplementary Figure S2. Hormone-induced *PR1*, *VSP2* and *PDF1-2a* gene expression monitored by qRT-PCR.** **(A-C)** qRT-PCR analysis of *PR1* (A), *VSP2* (B) and *PDF1-2a* (C) transcript levels in 12-day-old wildtype Col-0 after treatment for 1.0, 3.0, 6.0, 12, and 24 h with 0.5 mM SA, 50 µM MeJA and combination of MeJA with 2.0 µM ACC. The transcript abundance was normalized to the transcript level of the housekeeping gene *UBQ5*, and subsequently to the transcript level in mock. Data show means ± s.e.m. of three independent biological replicates, each representing a pool of 10 plants. The experiment was repeated once with a similar result. Asterisks indicate statistical differences between the transcript levels in hormone-treated and corresponding mock samples (*p < 0.05, **p < 0.01, ***p < 0.001, Student’s t-test). Different letters indicate the significant differences between groups (One-way ANOVA followed by Tukey’s multiple comparison test, p < 0.05).

**Supplementary Figure S3. Functional characterization of Arabidopsis COLORFUL-VSP2pro and –PDF1.2apro reporter lines. (A,B)** Reporter activities in the leaf pavement cells of four independent COLORFUL-VSP2pro (A) or –PDF1.2apro transgenic lines (B). **(C,D)** Effect of MeJA, and MeJA combined with ACC treatments on reference signal intensities in guard, pavement and palisade mesophyll cells of transgenic lines COLORFUL-VSP2pro#1 and COLORFUL-PDF1.2apro#1, respectively. (e-h) Reporter activities in the leaf pavement cells of transgenic lines COLORFUL-VSP2pro#1 and COLORFUL-PDF1.2apro#1 in response to dose **(E,G)** and incubation period **(F,H)** of MeJA, or MeJA combined with ACC treatment, respectively. Eleven-day-old seedlings were incubated in the respective hormones for 24 h, unless otherwise indicated. The experiments were repeated twice showing consistent results. Box plots show first quartile (lower line); median (center line); mean (+); third quartile (upper line); whiskers extend 1.5 times the interquartile range, and outliers (dots), n = 9-10. Data show means ± s.e.m. Data are relative to the mean value of mock of WT. **Different letters indicate significant differences (Two-way ANOVA followed by Tukey’s multiple comparison test, p < 0.05). Asterisks indicate significant differences (ns not significant, *p < 0.05, **p < 0.01, ***p < 0.001, one-tailed Student’s t-test).**

**Supplementary Figure S4. COLORFUL-PR1pro, -VSP2pro and –PDF1.2apro reporter activities in different Arabidopsis organs.** **(A-C)** Reporter activities in the epidermal pavement cells of leaves, cotyledons and root caps of 12-day-old transgenic lines COLORFUL-PR1pro#1 **(A)**, COLORFUL-VSP2pro#1 **(B)**, and COLORFUL-PDF1.2apro#1 **(C)**, after treatment with 0.5 mM SA, 50 µM MeJA and 50 µM MeJA + 0.2 µM ACC (red), respectively, for 24 hours in comparison to mocks (white). The experiments were repeated once showing similar results. Box plots show first quartile (lower line); median (centre line); mean (+); third quartile (upper line); whiskers extend 1.5 times the interquartile range, and outliers are depicted as dots, n = 7-10 samples. Data are relative to the mock of the wildtype. Different letters indicate the significant differences between groups (One-way ANOVA followed by Tukey’s multiple comparison test, p < 0.05).

**Supplementary Figure S5. Virulent (Noco2) and avirulent (Emwa1) isolates of *H. arabidopsidis* induce distinct whole-leaf hormone accumulation and signaling output patterns. (A-C) SA (A), JA (B) and JA-Ile (C) concentrations in the 5^th^ leave of three-week-old Arabidopsis Col-0 at 1 and 2 days post inoculation (dpi) with Noco2 and Emwa1. (D-F) Real-time qRT-PCR analysis of *PR1* (D), *VSP2* (E) and *PDF1.2* (F) transcript levels in the 5^th^ leaf of Arabidopsis Col-0 at 1 and 2 days post inoculation (dpi) with Noco2 and Emwa1. Data show means ± s.e.m. of three independent repeats, each represents a pool of 40-45 leaves. Different letters indicate significant differences (one-way ANOVA followed by Tukey’s multiple comparison test, p < 0.05).**

**Supplementary Figure S6. Dissection of cells associated with *H. arabidopsidis* (*Hpa*) invasion. (A)** Schematic representations depict the invasion dynamics of **virulent (Noco2) and avirulent (Emwa1) isolates of *Hpa* at** 1 and 2 day(s) post inoculation (dpi)**, and the** dissected cell zones at sites of invasion. **(B)** Representative Maximum projections of CLSM z-stack images used for quantification of COLORFUL-PR1pro responses at site of Emwa1 invasion. The Images show overlays of reporter (green), reference (magenta) and plasma membrane (gray) markers in leaf epidermis (left) and palisade mesophyll (right) of three-week-old Arabidopsis line COLORFUL-PR1pro#1 at 2 dpi after staining Emwa1 spore with FB28 (cyan). Arrowheads indicate Emwa1 invading structures. Invaded cells (orange discontinuous lines), adjacent cells (yellow discontinuous lines) and distant cells (not highlighted). Scale bar, 25 μm.

**Supplementary Figure S7. COLORFUL-PR1pro, -VSP2pro and –PDF1.2apro reporter signaling signatures at Arabidopsis-*H. arabidopsidis* interaction sites.** COLORFUL-PR1pro **(A,B)**, -VSP2pro **(C,D)** and –PDF1.2apro **(E,F)** reporter activities in pavement cells (top) and palisade mesophyll cells (bottom) at the sites of invasion by virulent (Noco2) and avirulent (Emwa1) isolates of *Hpa* at 1 (A,C,E) and 2 (B,D,F) days post inoculation (dpi). The experiments were repeated twice showing consistent results. Box plots show first quartile (lower line); median (center line); mean (+); third quartile (upper line); whiskers extend 1.5 times the interquartile range, and outliers (dots), n = 6-9. Data show means ± s.e.m. Data are relative to the mean value of mock of WT. **Different letters indicate significant differences (Two-way ANOVA followed by Tukey’s multiple comparison test, p < 0.05). Asterisks indicate significant differences (ns not significant, *p < 0.05, **p < 0.01, ***p < 0.001, one-tailed Student’s t-test).**
