# Supplementary material for: Quantitative Hormone Signaling Output Analyses of Arabidopsis thaliana Interactions With Virulent and Avirulent Hyaloperonospora arabidopsidis Isolates at Single-Cell Resolution
Source: Front Plant Sci. 2020 Nov 6;11:603693. doi: 10.3389/fpls.2020.603693 (PMC7677359; doi:10.3389/fpls.2020.603693)
Supplement: Supplementary Method 1 — Determination of Phytohormones by UPLC-nano ESI-MS/MS. [file Data_Sheet_2.DOC]

**Supplementary Materials and Methods**

**Determination of phytohormones by UPLC-nano ESI-MS/MS**

Extraction was performed as previously described for lipids, with some modifications . Plant material (100 mg) were extracted with 0.75 mL of methanol containing 10 ng D_4_-SA, 10 ng D_5_-JA (both from C/D/N Isotopes Inc., Pointe-Claire, Canada), 10 ng D_4_-JA-Leu (kindly provided by Otto Miersch, Halle/Saale, Germany), each as internal standard. After vortexing, 2.5 mL of methyl-*tert*-butyl ether (MTBE) were added and the extract was shaken for 1 h at room temperature. For phase separation, 0.6 mL water were added. The mixture was incubated for 10 min at room temperature and centrifuged at 450 x g for 15 min. The upper phase was collected and the lower phase was re-extracted with 0.7 mL methanol/water (3:2,5, v/v) and 1.3 mL MTBE as described above. The combined upper phases were dried under streaming nitrogen and resuspended in 100 μl of acetonitrile/water (20:80, v/v) containing 0.3 mmol/l NH_4_HCOO (adjusted to pH 3.5 with formic acid).

Reversed phase separation of constituents was achieved by UPLC using an ACQUITY UPLC® system (Waters Corp., Milford, MA, USA) equipped with an ACQUITY UPLC® HSS T3 column (100 mm x 1 mm, 1.8 µm; Waters Corp., Milford, MA, USA). Aliquots of 10 µl were injected in a partial loop with needle overfill mode. Elution was adapted to . Solvent A and B were water and acetonitrile/water (90:10, v/v), respectively, both containing 0.3 mmol/l NH_4_HCOO (adjusted to pH 3.5 with formic acid). The flow rate was 0.16 ml/min and the separation temperature was constantly at 40 °C. Elution was performed isocratically for 0.5 min at 10% solution B, followed by a linear increase to 40% solution B in 1.5 min, this condition was held for 2 min, followed by a linear increase to 95% solution B in 1 min, this condition was held for 2.5 min. The column was re-equilibrated for start conditions in 3 min.

Nanoelectrospray (nanoESI) analysis was achieved using a chip ion source (TriVersa Nanomate®; Advion BioSciences, Ithaca, NY, USA). For stable nanoESI, 70 µl min^-1^ of 2-propanol/acetonitrile/water (70:20:10, v/v/v) containing 0.3 mmol/l NH_4_HCOO (adjusted to pH 3.5 with formic acid) delivered by a Pharmacia 2248 HPLC pump (GE Healthcare, Munich, Germany) were added just after the column via a mixing tee valve. By using another post column splitter 502 nl min^-1^ of the eluent were directed to the nanoESI chip with 5 µm internal diameter nozzles. Ionization voltage was set to -1.7 kV. Phytohormones were ionized in a negative mode and determined in scheduled multiple reaction monitoring mode with an AB Sciex 4000 QTRAP® tandem mass spectrometer (AB Sciex, Framingham, MA, USA). Mass transitions were as previously described , with some modifications and were as follows: 214/62 [declustering potential (DP) ‑35 V, entrance potential (EP) ‑8.5 V, collision energy (CE) ‑24 V] for D_5_-JA, 209/59 (DP ‑30 V, EP ‑4.5 V, CE ‑24 V) for JA, 325/133 (DP ‑65 V, EP ‑4 V, CE ‑30 V) for D_4_-JA-Leu, 322/130 (DP ‑45 V, EP ‑5 V, CE ‑28 V) for JA-Ile, 141/97 (DP ‑25 V, EP ‑6 V, CE ‑22 V) for D_4_-SA and 137/93 (DP ‑25 V, EP ‑6 V, CE ‑20 V) for SA. The mass analyzers were adjusted to a resolution of 0.7 amu full width at half-height. The ion source temperature was 40 °C, and the curtain gas was set at 10 (given in arbitrary units). Quantification was carried out using a calibration curve of intensity (m/z) ratios of [unlabeled]/[deuterium-labeled] vs. molar amounts of unlabeled (0.3-1000 pmol).

Balcke, G.U., Handrick, V., Bergau, N., Fichtner, M., Henning, A., Stellmach, H., Tissier, A., Hause, B., and Frolov, A. (2012). An UPLC-MS/MS method for highly sensitive high-throughput analysis of phytohormones in plant tissues. *Plant methods* 8**,** 47

Iven, T., König, S., Singh, S., Braus-Stromeyer, S.A., Bischoff, M., Tietze, L.F., Braus, G.H., Lipka, V., Feussner, I., and Dröge-Laser, W. (2012). Transcriptional activation and production of tryptophan-derived secondary metabolites in Arabidopsis roots contributes to the defense against the fungal vascular pathogen Verticillium longisporum. *Molecular Plant* 5**,** 1389-1402

Matyash, V., Liebisch, G., Kurzchalia, T.V., Shevchenko, A., and Schwudke, D. (2008). Lipid extraction by methyl-tert-butyl ether for high-throughput lipidomics. *Journal of lipid research* 49**,** 1137-1146
